# Retinal waves align the concentric orientation map in mouse superior colliculus to the center of vision

**DOI:** 10.1126/sciadv.adf4240

**Published:** 2023-05-12

**Authors:** Kai Lun Teh, Jérémie Sibille, Carolin Gehr, Jens Kremkow

**Affiliations:** ^1^ Neuroscience Research Center, Charité-Universitätsmedizin Berlin, Charitéplatz 1, Berlin 10117, Germany.; ^2^ Bernstein Center for Computational Neuroscience Berlin, Philippstraße 13, Berlin 10115, Germany.; ^3^ Institute for Theoretical Biology, Humboldt-Universität zu Berlin, Philippstraße 13, Berlin 10115, Germany.; ^4^ Einstein Center for Neurosciences Berlin, Charitéplatz 1, Berlin, Germany.

## Abstract

Neurons in the mouse superior colliculus (SC) are arranged in a concentric orientation map, which is aligned to the center of vision and the optic flow experienced by the mouse. The origin of this map remains unclear. Here, we propose that spontaneous retinal waves during development provide a scaffold to establish the concentric orientation map within the SC and its alignment to the optic flow. We test this hypothesis by modeling the orientation-tuned SC neurons that receive ON/OFF retinal inputs. Our model suggests that the propagation direction bias of stage III retinal waves, together with OFF-delayed responses, shapes the spatial organization of the orientation map. The OFF delay establishes orientation-tuned neurons by segregating their ON/OFF receptive subfields, the wave-like activities form the concentric pattern, and the direction biases align the map to the center of vision. Together, retinal waves may play an instructive role in establishing functional properties of single SC neurons and their spatial organization within maps.

## Teaser

Developmental retinal waves establish an optic-flow-aligned orientation map in the mouse superior colliculus.

## INTRODUCTION

**Fig. 1.**
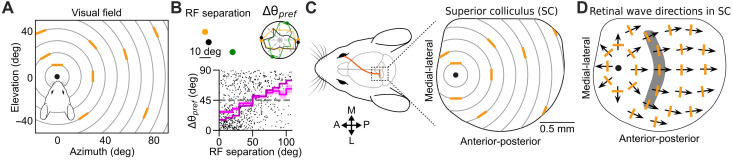
Hypothesis: Concentric OPM is shaped by the retinal waves during development. (**A**) Orientation preference of SC neurons (orange bars) plotted in different parts of the visual field. The orientation preferences in the SC are organized in a concentric pattern around the center of vision (nose position). Gray circles indicate the concentric angles, which are orthogonal to the radial angles, plotted continuously at a fixed distance from the center of vision (black dot). Data adapted from (*8*). (**B**) In vivo tangential electrophysiological recordings in the mouse SC. Top: Individual examples showing that the neurons with close-by RFs (orange and black dots) have similar orientation preferences than a neuron with a further RF (green dot). The polar plot shows the responses of the SC neurons to moving gratings. The dots located on the boundary of the polar plot indicate the orientation preferences of the neurons with corresponding colors. Bottom: Absolute difference in orientation preferences against the RF separation of the SC neurons (*n* = 5 mice, 78 neurons, 742 pairs). The magenta line indicates the means for 10° bins ± SEM; the purple line indicates the medians for 10° bins, and the gray dashed line indicates the absolute difference expected by chance (45°). (**C**) Retinotopic mapping of the orientation preferences shown in (A) to the mouse SC. (**D**) Retinal waves during development. The radial retinal wave flow pattern with its center (black dot) located at the anterior part of the SC. Arrows represent the net wave vectors, which indicate the local wave directional bias. The local retinal wave vectors resemble the optic flow (*13*) but are orthogonal to the concentric orientation preferences (orange bars).

The superior colliculus (SC) is a midbrain structure that is important for visually guided behaviors. The neurons in the mouse SC are selective for stimulus attributes like orientation, direction, and ON/OFF polarity (*1*–*7*). The representations of these stimulus features are arranged in functional maps, such as the orientation preference map (OPM) (*8*, *9*) and direction preference map (*10*, *11*) [but see (*12*)]. The SC OPM is organized in a concentric pattern centered around the nose position (Fig. 1, A and C) (*8*), reminiscent of the optic flow experienced by the animals (*13*). However, the mechanisms that give rise to the concentric OPM in the SC remain elusive.

The main afferent input to the superficial SC arises from retinal ganglion cells (RGCs), the output neurons of the retina. There is growing evidence that the topographic organization in the SC emerged during the developmental period (*13*, *14*). During development of the visual system, spontaneously evoked activities, termed retinal waves, are spreading across the developing retinas. The retinal waves have been shown to play important roles in refining the neural circuits of the visual systems (*15*). Specifically, the stage II (S2) retinal waves are responsible for eye-specific segregation and gross retinotopic refinement (*16*, *17*), whereas the stage III (S3) retinal waves are known for ON/OFF segregation in the retinas and fine-scale retinotopic refinement (*18*–*20*). However, it is largely unknown whether retinal waves also play a role in establishing the functional maps in the SC.

During development, retinal waves strongly drive the SC activity (*14*, *21*). The retinal waves do not propagate in a random manner across the retina but show a clear bias in their propagation directions. More specifically, the waves tend to propagate in the temporal-to-nasal directions in the retina, corresponding to the anterior-to-posterior directions in the SC (*13*, *14*, *21*). When mapped onto the SC surface, the net wave flow pattern, which indicates the net local bias of the wave propagation directions, resembles the optic flow experienced by the animals (*13*) and matches the concentric OPM observed in vivo (Fig. 1D) (*8*). While both S2 and S3 waves show direction biases to some extent, only S3 waves have distinct ON/OFF responses, where the ON and OFF RGCs are activated asynchronously, with OFF RGCs having a delay relative to ON RGCs (*22*). This asynchronous ON/OFF activation results in the coactivation of ON and OFF RGCs from neighboring areas, providing a plausible mechanism for establishing orientation-tuned receptive field (RF) in the postsynaptic SC neurons (*23*). Therefore, we hypothesize that the concentric pattern is globally coordinated by the wave propagation direction biases, and the orientation preference of the SC neurons is locally established by the OFF delay. That is, we propose that the S3 retinal waves play a role in shaping the SC OPM during development. Our hypothesis predicts that (i) the wave propagation direction biases provide a scaffold for the formation of functional maps, and (ii) the OFF delay plays a role in shaping the RF of the postsynaptic SC neurons.

**Fig. 2.**
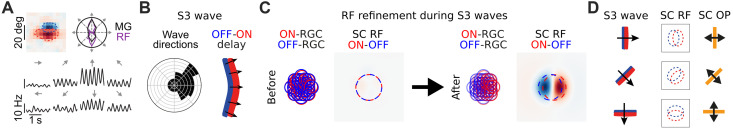
Computational model of retinocollicular circuit development. (**A**) Top: Example SC neuron measured in vivo with ON/OFF RF structure (left) that matches its responses to the moving-grating (MG) stimuli (right). The red and blue dashed lines indicate the contours of the ON and OFF subfields, respectively. The orientation tuning predicted from the Fourier transform of the RF (purple) is consistent with the mean MG responses (black). Bottom: Responses of the neuron to MG stimuli of eight different directions. (**B**) Developmental mechanism underlying the ON/OFF segregation. During S3 waves, the wave propagation direction bias (left) and the delayed OFF-RGC responses (right) result in the net coactivation of the ON and OFF RGCs in the area covered by the waves, with the ON wave leading the OFF wave. (**C**) Refinement of the RF of postsynaptic SC neurons via Hebbian learning during S3 waves. Before the refinement process, the input strengths from the ON and OFF RGCs to a postsynaptic SC neuron are identical everywhere within the arbor range. This results in the completely overlapped ON/OFF RGC inputs to the SC neuron, which gives little to no net ON/OFF subfields in the postsynaptic SC neuron. The S3 waves then gradually remold the RGC input strengths via Hebbian learning. We hypothesize that, after S3 waves, the net local wave activities due to the propagation direction bias will locally shape the RGC input strengths such that the strongest ON and OFF inputs are deviated and aligned to the net local wave direction. Left: Weighted RGC RFs. Right: Resulting SC RF. The dashed lines indicate the contour for the ON (red) and OFF (blue) subfields. (**D**) The orientation preference (OP) of the SC neuron (right) depends on the configuration of the ON and OFF receptive subfields (middle), which in turn depends on the net local bias of the retinal wave direction (left).

In this work, we test this hypothesis using a computational model (Fig. 2, B to D) and systematically analyze the effects of these two S3 wave properties. With our model, we show that wave-like activities naturally give rise to a radial wave flow pattern, which in turn facilitates the establishment of a concentric OPM. We also found that a proper OFF delay could facilitate the development of well-tuned RFs. Last, our model discovers a surprising role of the wave propagation direction biases in aligning the OPM to the concentric angles of the visual field centered at the putative nose position (center of vision: 0° azimuth and 0° elevation), providing a promising mechanism for explaining the observations by Ahmadlou and Heimel (*8*). Together, our model outlines the interplay between seemingly unrelated developmental mechanisms in shaping the SC OPM, thus providing a direct mapping from developmental features to functions in the SC. In other words, our findings support the notion that the retinal waves instruct the formation of the functional circuits in the SC.

## RESULTS

Using our existing dataset of in vivo tangential electrophysiological recordings in anesthetized mice (*24*, *25*), we observed that there is a tendency for the orientation-tuned SC neurons with RF in close proximity to have similar orientation preferences (Fig. 1B), consistent with the observations from Ahmadlou and Heimel (*8*) and Feinberg and Meister (*9*). The SC neurons receive afferent inputs from a variety of RGC types. Depending on the functional type, the RGC axonal arbors are organized in different SC layers (*26*). Among the RGC types that project to SC are the classical ON-center and OFF-center RGCs that are not orientation tuned, and which drive ON and OFF responses in SC neurons (*7*). The axons of these ON/OFF RGCs terminate in the lower layers of the superficial SC, namely, the lower stratum griseum superficiale (SGS) and stratum opticum (SO) layers (*26*–*28*). A recent study has shown that the ON and OFF retinal inputs can converge onto individual SC neurons (*24*). Here, we model the convergence of the ON-center and OFF-center RGC inputs in the lower SGS and SO layers for establishing the orientation preference of SC neurons (Fig. 2), similar to how the convergence of ON/OFF inputs established tuning properties in other parts of the visual system (*29*–*31*). This assumption is in line with results showing that while direction-selective neurons are concentrated in the upper SGS of the superficial SC (*4*, *32*), orientation-selective neurons are more evenly distributed across all SC layers, including the superficial (upper SGS, lower SGS, and SO) and deep (layers below the SO) SC, with the linear orientation–selective neurons almost exclusively present in the superficial SC (*32*). Furthermore, our in vivo data suggest that there is a subset of SC neurons that are orientation tuned and have ON/OFF RF structure matching their orientation preference existing in the superficial SC (Fig. 2A and fig. S1). We further assume that the spatial organization of the RFs is linked to the statistics of the retinal waves during development such that retinal waves shape the segregation of ON/OFF inputs in a refinement process. This assumption is supported by results showing that retinal waves are important for the refinement of the RGC axons during development (*16*, *27*, *33*).

Subsequently, we ask what could be the underlying mechanisms that refine the RF of SC neurons and establish orientation preference during development. It has been reported that the ON- and OFF-RGC activities during S3 waves are temporally anti-correlated where OFF RGCs are active with a delay of ⁓1 s relative to the ON RGCs (Fig. 2B, right) (*22*). This asynchronous pattern of ON/OFF RGC activation has been proposed to play a role in ON/OFF segregation in the lateral geniculate nucleus (*22*, *34*). However, its role in the SC is still unclear. Here, we propose that this OFF delay plays an instructive role in establishing SC orientation preferences by providing a spatial template that selectively strengthens the ON inputs from the leading ON-wave region and OFF inputs from the following OFF-wave region with little overlap, thereby segregating the ON and OFF subfields of the SC neurons (Fig. 2, B and C). Hebbian learning with subtractive normalization (*35*–*38*) was implemented for strengthening the coactivated presynaptic RGC inputs to the postsynaptic SC neurons (Fig. 2C). In this framework, the OFF delay, together with the bias in wave propagation direction, establishes the orientation preference by fine-tuning the spatial organization of the ON/OFF subfields (Fig. 2D).

In summary, the model links the retinal wave propagation and OFF delay via Hebbian learning to the RF structure of the SC neurons. In the following, we will first demonstrate how the RF structure of local single SC neurons is dependent on the OFF delay and the local wave propagation direction bias. After that, we will show how these wave features give rise to a smooth SC OPM and how their global statistics affect the map properties.

### OFF delay and local wave directional bias establish well-tuned SC neurons

**Fig. 3.**
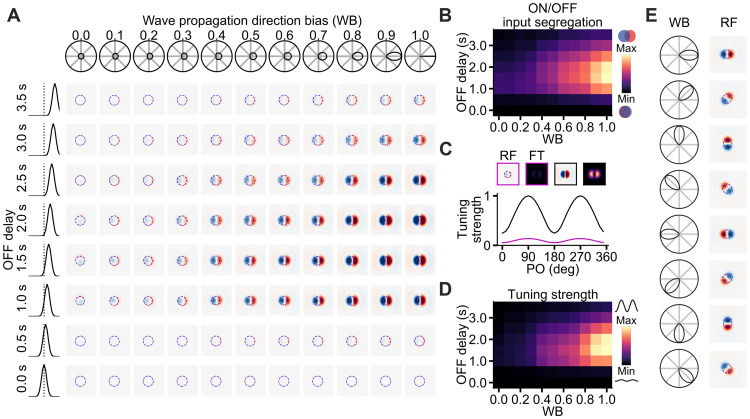
ON/OFF retinal waves shape the RF properties of SC neurons. (**A**) Effects of OFF delay and net local wave directional bias on the RF. The OFF delay of the S3 waves is needed for the separation of the ON and OFF receptive subfields. As long as there is OFF delay, higher local wave directional bias gives more prominent RF, where the OFF delays of 1.5 and 2 s give the most prominent RF. Leftmost column: OFF-to-ON cross-correlogram for 0 s (bottom) to 3.5 s OFF delays (top). Topmost row: Polar probability distribution of the wave directions corresponding to the bias indices. (**B**) Corresponding ON/OFF input segregation for the RFs in (A). (**C**) Tuning curves of two RFs with wave propagation direction biases of 0.1 (purple) and 1 (black), both with OFF delay of 1.5 s. Insets are the corresponding RFs and their Fourier transform (FT). (**D**) Corresponding tuning strengths for the RFs in (A). (**E**) The net local wave directional bias determines the final configuration of the ON and OFF receptive subfields, where the ON subfield is leading the OFF subfield in the direction of the net wave vector. Red/blue dashed lines: Contour lines for ON/OFF receptive subfields.

To start examining the role of the net local retinal wave vector in establishing the RF properties of the postsynaptic SC neurons, we systematically varied the OFF delay (τ_OFF_) from 0 to 3.5 s and the retinal wave propagation direction bias from 0 (no bias, all directions equally probable) to 1 (only waves with the same direction) (Fig. 3A). Retinal waves propagating from eight equally spaced directions toward the center with biases in the range [0,1] were implemented to establish the RF of the central SC neuron. The activity of the RGCs was simulated with a response kernel with a constant firing rate (Eq. 9). For overlapping ON/OFF waves (τ_OFF_ = 0 s), the SC RF failed to segregate into ON and OFF subfields, even with high wave propagation direction biases (Fig. 3, A and B). This could be explained by the Hebbian mechanism as under this configuration both ON and OFF inputs at the same spatial location will increase their strength together and therefore cancel out each other when converging on the SC neuron. The segregation of the ON and OFF subfields, which is established by the spatial segregation of the ON/OFF inputs (fig. S4), emerged once there is an OFF delay (Fig. 3B). The optimal OFF delay is around 1 to 2 s, which is consistent with the experimental observations (*22*). Thus, the OFF delay during the S3 waves is essential for segregating the ON/OFF subfields of SC neurons during development. However, what controls the spatial configuration of the ON/OFF subfields? When the local retinal wave directions are uniformly distributed, the SC RF failed to develop strong ON/OFF subfields even with optimal OFF delays (Fig. 3A, first column of RFs). Increasing the local wave directional bias increases the SC RF contrast and is maximum for an OFF delay of around 1 to 2 s and for retinal waves with strong directional bias.

The ON/OFF segregation and orientation tuning strength of the RF have the same trend where the segregation and the tuning strength increase as the wave directional bias increases and the optimal OFF delay occurred at around 1 to 2 s (Fig. 3, B and D). The optimal OFF delay for the RF contrast, ON/OFF segregation, and tuning strength is dependent on the size of the integration site (Eq. 14), where a smaller integration site will result in a shorter optimal OFF delay (fig. S3). Figure 3C compares two tuning curves obtained from the Fourier transform of the corresponding RFs (Eq. 29) established with different wave directional biases.

To test whether our model could generate orientation-tuned SC neurons to any direction of retinal waves, we simulated the retinal waves with bias to eight different directions. As expected, the SC ON/OFF subfields arranged to the direction of the net wave vector, with the ON subfield leading the OFF subfield (Fig. 3E). This shows that the OFF delay will result in the coactivation of neighboring ON and OFF RGC inputs. Therefore, with the Hebbian mechanism, the OFF delay helped segregate and establish the ON and OFF receptive subfields of the postsynaptic SC neurons, which in turn determined the resulting orientation preference of those SC neurons (Fig. 3E, right). Thus, this proposed model could establish orientation-tuned RFs in the postsynaptic SC neuron with spatial configuration shaped by the direction of the net local wave vector. Together, biological realistic values of the OFF delay (1 to 2 s) observed during S3 retinal waves with sufficient local wave directional bias can establish a well-tuned RF in the SC neuron, with the spatial organization of the RF arranged in the biased wave direction.

### Radial wave flow pattern gives rise to concentric map organization

We propose that the OPM in the SC is determined by the pattern of the net retinal wave flow. This hypothesis is supported by the observations that the S2 and S3 retinal waves do not propagate uniformly to all directions (*14*, *21*), and a net wave flow pattern has been measured (*13*). This net wave flow pattern indicates the net excess wave activities in a direction during these developmental stages, which could potentially shape the functional organization of the target structures. As shown in the previous section, these wave directionality–induced local excessive activities could have an impact on the RGC-SC connections and give rise to an orientation-tuned SC RF. Now, we are going to explore the effects of these wave properties, namely, the OFF delay and the wave propagation direction bias, on the overall organization of the SC OPM.

**Fig. 4.**
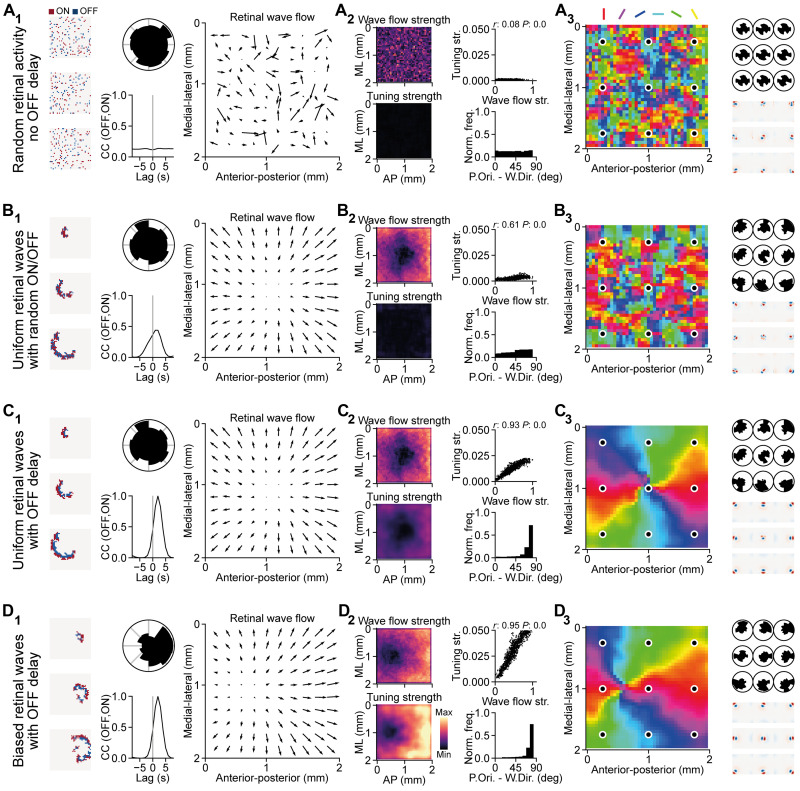
Wave-like activities with OFF delay establish a smooth SC OPM. (**A**) Random retinal activities. (**B**) Uniform retinal waves with random ON/OFF activation. (**C**) Uniform retinal waves with OFF delay. (**D**) Biased retinal waves with OFF delay. (A_1_ to D_1_) Left: Examples of retinal waves. Middle: Distribution of wave propagation directions (top) and the cross-correlogram of the OFF relative to ON RGC activities (bottom). Right: Retinal wave flow. (A_2_ to D_2_) Left: Strength of the retinal wave flow (top) that reflects the local bias of wave propagation directions and the orientation tuning strength (bottom). Right: Relationship between the wave flow strength and the orientation tuning strength (top) and distribution of absolute angular difference between the orientation preference and the wave propagation direction (bottom). Note that the retinal waves with OFF delay give rise to orthogonality between the orientation preference and the wave propagation direction (C_2_ and D_2_). (A_3_ to D_3_) Left: Resulting OPM. Right: Distribution of local wave propagation directions (top) and RFs (bottom) corresponding to the nine locations marked in the OPM.

Non–wave-like retinal activities without consistent OFF delay (Fig. 4, A_1_, left) resulted in random wave vectors (Fig. 4, A_1_, right) and thus did not produce a smooth OPM (Fig. 4, A_3_, left). Similarly, retinal waves with uniform propagation directions but with random ON/OFF activation also did not give rise to a smooth OPM (Fig. 4, B_3_, left), despite having smooth wave vectors (Fig. 4, B_1_, right). This indicates that the ON/OFF activation pattern (i.e., the OFF delay), and not just the wave-like activities, is also important in establishing a smooth OPM.

Next, we tested the model with uniform retinal waves (Fig. 4, C_1_, top middle) with τ_OFF_ = 1 s to see its effects. To our surprise, these retinal waves without propagation direction biases already gave rise to a smooth concentric OPM with its singularity located at the center of the model (Fig. 4, C_3_, left), matching the center of the radial wave flow pattern (Fig. 4, C_1_, right). A possible explanation is that although there was no global wave propagation direction bias (Fig. 4, C_1_, top middle), there were still local biases at every retinotopic location (Fig. 4, C_3_, top right), which formed a radial wave flow pattern (Fig. 4, C_1_, right). Last, the retinal waves with biased propagation in the anterior-to-posterior directions (which is temporal-to-nasal in the retina) and τ_OFF_ = 1 s (Fig. 4, D_1_, top middle) produced smooth wave vectors that formed a radial pattern (Fig. 4, D_1_, right) and smooth OPM with its singularity shifted toward the anterior SC (Fig. 4, D_3_, left). This result is consistent with the concentric OPM observed in vivo (*8*).

Therefore, our results indicate that the wave-like retinal activities can produce wave vectors that are changing gradually across neighboring retinotopic locations and have strong magnitude (Fig. 4, B_2_ to D_2_, top left). With proper ON/OFF temporal structure, these wave vectors that represent local wave directional bias can give rise to RFs with strong tuning strengths (Fig. 4, C_2_ and D_2_, bottom left) and thus producing a smooth OPM. The resulting orientation preferences are arranged orthogonally to the directions of these net local wave vectors (Fig. 4, C_2_ and D_2_, bottom right). In other words, the net wave flow that has a radial pattern determines the globally concentric organization of the OPM.

### Globally biased retinal waves align the concentric map to the visual field center

**Fig. 5.**
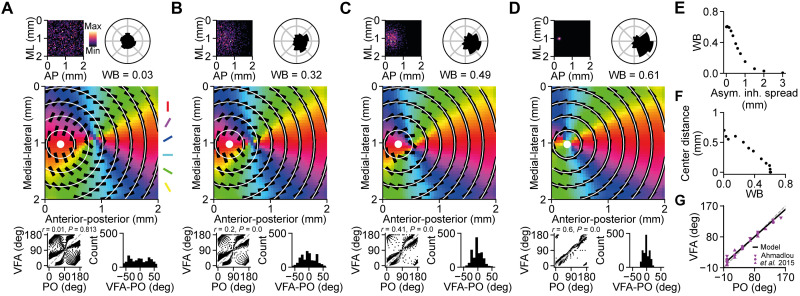
Small asymmetric inhibition spread creates a bias in the retinal waves that aligns the OPM to the concentric angles of the visual field. (**A** to **D**) Top: Distribution of the source positions of asymmetric inhibition (left) and wave direction distribution (right). Middle: Overlay of concentric angles of the visual field (circular lines) on the OPM. With higher wave direction biases, the local orientation preferences (black bars) are more aligned to the concentric visual field angles. The white dot indicates the visual field center. Bottom: Concentric visual field angles against the orientation preference (left) and the frequency distribution of their angular differences (right). (**E**) Relationship between the wave propagation direction biases and the spreads of the asymmetric inhibition distribution. Smaller asymmetric inhibition spreads give higher bias in the wave propagation directions. (**F**) The distance between the visual field center and the OPM singularity decreases as the wave direction bias increases. (**G**) The model with wave direction bias of 0.61 is consistent with the in vivo observation. Data replotted from (*8*).

In the previous section, we showed that the wave propagation direction biases could affect the position of the singularity in the concentric OPM, but what is the underlying mechanism? In our model, retinal waves propagated away from a source of asymmetric inhibition (fig. S2), which is inspired by the asymmetric inhibition mediated by the starburst amacrine cells observed in vivo (*13*). For each wave initiation, the source of asymmetric inhibition was sampled from a two-dimensional (2D) Gaussian distribution centered at the center of vision (predefined nose position). The wave directional bias decreases as the spread of the asymmetric inhibition distribution increases (Fig. 5E). Thus, the location and spread of the asymmetric inhibition could play a key role in shifting the map singularity.

To systematically study the role of the asymmetric inhibition on the location of the OPM singularity, we gradually decreased the spread of the asymmetric inhibition distribution (Fig. 5, A to D, top left). As expected, the location of the singularity gradually changed from the center of the SC toward the center of the asymmetric inhibition distribution. Shifting the center of an asymmetric inhibition distribution with a small spread could also shift the singularity of the resulting OPM (fig. S5) and change the bias of the resulting wave direction frequency distribution (fig. S6D). The relationships among the asymmetric inhibition distribution, wave initiation map, and wave direction frequency distribution are illustrated in fig. S6.

However, instead of changing the center position of the asymmetric inhibition distribution, we fixed its center at the center of vision and changed its spread from 2000 μm (Fig. 5A), which is equivalent to a uniform distribution that has no overall asymmetric inhibition, to 50 μm (Fig. 5D), which gives high asymmetric inhibition originating from the visual field center. Nevertheless, in both cases, the highly localized sources of asymmetric inhibition around the visual field center are the key factor that gives rise to wave direction biases and thus shifts the OPM singularity toward the visual field center (Fig. 5, D and F). Misalignment between the OPM and the visual field will result in a large angular difference between the orientation preferences and the concentric angles of the visual field (Fig. 5, A and B, bottom). The waves with direction bias of around 0.61 resulted in an angular difference distribution similar to in vivo observation (Fig. 5D) (*8*). Figure 5G shows the comparison between the model and the data from Ahmadlou and Heimel (*8*) on the concentric visual field angle against the preferred orientation.

### Robustness of the concentric map pattern to the activity noise

**Fig. 6.**
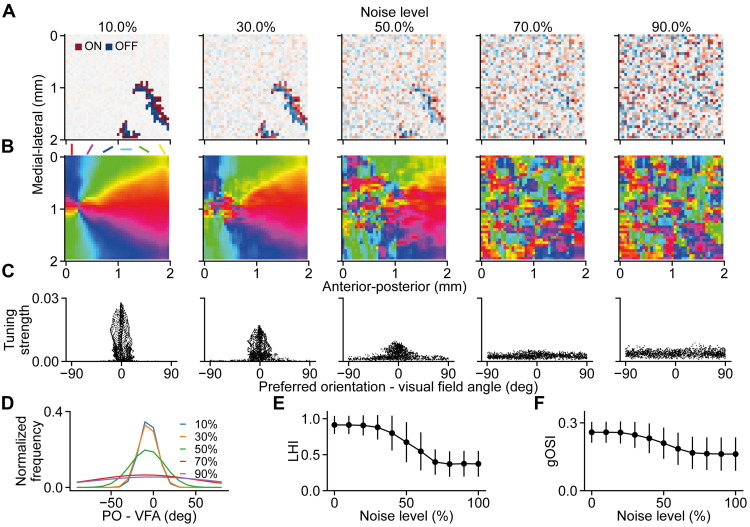
Robustness of the model to activity noise. (**A**) Five examples of wave frames with different levels of activity noise. (**B**) The resulting OPMs become less and less smooth as the activity noise increases. Note that the OPM singularity gets affected first as the noise level increases. (**C**) Tuning strengths versus the angular difference between the preferred orientation and the concentric visual field angle. As the noise level increases, the tuning strength decreases and the angular difference becomes larger. (**D**) The distribution of the angular difference between the preferred orientation and the concentric visual field angles remained unchanged up to 30% activity noise level. (**E**) LHI decreases as the noise level increases. (**F**) gOSI decreases slightly as the noise level increases. Error bars indicate the SD.

Is the proposed model sufficiently robust to background activity noise? To answer this question, we tested the model with different fractions of signal (the waves) and noise (the random background activities), which are determined by *r* and γ in Eq. 9, respectively, where *r* + γ = 1 and *r*, γ ≥ 0. With increasing fraction of noise, the waves became less and less prominent (Fig. 6A). As expected, the resulting OPM became less smooth with increasing noise level (Fig. 6B). An interesting observation is that the area around the singularity of the OPM got affected first by the noise, which could be explained by the relatively low wave flow strength around the map singularity (Fig. 4, C_2_ and D_2_).

In addition, as the noise level increases, the orientation tuning strength decreases, and the angular difference between the preferred orientation and the concentric visual field angle becomes larger (Fig. 6C). Up to 30% noise level, the angular difference remained largely intact (Fig. 6D) despite the decrease of the overall orientation tuning strength (Fig. 6C).

To quantify the effects of the noise on the map properties, we computed the local homogeneity index (LHI) (*39*) and the global orientation selectivity index (gOSI). As shown, the model is robust up to 30% activity noise level with little effects on LHI and gOSI (Fig. 6, E and F). Above 30% noise level, LHI and gOSI started to decrease steeply and reached the minimum at 70% noise level.

## DISCUSSION

How does the concentric OPM arise in the mouse SC and why is it aligned to the center of vision? Here, we advance the notion that the SC OPM could be an emergent phenomenon that originates from retinal waves during development. The two key findings of our study are (i) the wave-like activities naturally give rise to a radial organization of the net wave flow field, which in turn facilitates the emergence of a concentric OPM (Fig. 4), and (ii) the wave directionality shifts the center of the radial net wave flow, and therefore the singularity of the OPM toward the center of the asymmetric inhibition sources (Fig. 5). Together, these two mechanisms provide an elegant explanation for the retinotopic-based orientation preference (*8*, *9*) and globally concentric organization (*8*) in the SC OPM. Since S3 retinal waves tend to propagate toward the posterior SC (*13*, *14*), which implies that the source of asymmetric inhibition is located at the anterior SC that corresponds to the nose position in the visual field, this suggests that the retinal wave directionality plays a role in aligning the functional maps to the optic flow, supporting the findings of our model. This alignment of the singularity of the OPM to the wave flow center at the anterior part of the SC could explain the lack of a map singularity in the observation by Feinberg and Meister (*9*) as they were measuring at the posterior SC.

A possible explanation for the emergence of a radial organization of the net wave flow is that the continuous spatiotemporal structure of a wave will activate neighboring RGCs simultaneously, resulting in smooth continuous change of the net local wave vectors in neighboring areas. Application of gabazine in the retina during development, which interferes with the asymmetric inhibition that underlies the wave direction bias of S3 waves, affects the direction selectivity of SC neurons but has little effect on SC neurons that are orientation tuned (*13*). Our model can recapitulate this finding since a concentric OPM was still established during unbiased waves (Figs. 4 and 5), just with a mismatch of the visual field center and the OPM singularity, which could be easily tested experimentally.

What could be the potential functional roles of aligning the concentric SC OPM to the center of vision? Recently, Ge *et al.* (*13*) showed that the pattern of retinal waves during development matches the optic flow experienced by the adult animals. Thus, following this idea, a potential role of the concentric OPM could be important for the processing of the orientations of visual objects experienced during natural behaviors. However, it remains to be shown what benefit the match of the visual field center and the SC OPM singularity brings for the perception and visually guided behaviors of the animal. On the other hand, the distribution of tuning strengths on the established OPM might have more obvious behavioral implications. The tuning strengths become higher as the distance to the map singularity increases (Fig. 4, C_2_ to D_2_). That is, the orientation tuning strengths are the lowest at the singularity and the highest at the periphery of the OPM. This means that the SC is likely to be more sensitive to the orientation- and motion-related stimuli at the periphery of the visual field that are important for the detection of potential dangers and initiation of defensive behaviors. Further experiments have to be conducted to confirm this prediction.

The use of Hebbian learning to refine the RGC-SC connections, which requires strong coactivation between RGCs and SC neurons, is supported by the experimental work showing that retinal waves strongly drive SC neurons during S3 waves (*14*). Besides, Hebbian-like mechanisms were shown to be at play in the developing retinocollicular synapses (*40*, *41*) and even in the shaping of postsynaptic RF (*42*). This suggests that the Hebbian mechanisms could be crucial in forming the functional maps in the SC. We further showed that the concentric OPM formed with Hebbian mechanisms is robust against the noise induced during the synaptic weight update (fig. S8), and the disruptive effects of the noise could be mitigated with a larger number of waves (fig. S8, G and H). Similarly, the dropout of synaptic update used to mimic the diminished plasticity at the later stage of development (*41*) has little effect on the OPM properties (fig. S9), and the most affected property, the tuning strength, can also be rescued with a larger number of waves (fig. S9I). Given that there are around 5 days of S3 waves before eye opening (*15*) with an average frequency of eight waves per minute (*14*), the total number of S3 waves before eye opening is estimated to be around 50,000 to 60,000, which should be sufficiently large for the formation of an SC OPM even with plasticity noise and diminished plasticity toward the later stage of the development (*41*). On the other hand, Gribizis *et al.* (*14*) also showed that the activities of the primary visual cortex (V1) do not correlate strongly with the S3 retinal waves, providing a plausible explanation for the salt-and-pepper organization in the mouse V1 that is different from the concentric SC OPM. These observations suggest that the retinocollicular and thalamocortical circuits may use different developmental mechanisms for establishing the functional organization. Consistent with this idea, we recently showed that RGC axons form mosaics in the SC that is isomorphic to the retinal mosaic (*24*), while, in contrast, thalamocortical axons seem to form clusters in the V1, both in species with and without OPM (*31*, *43*). This could be related to how visual information is represented in these two different circuits; however, more work needs to be done to better understand this.

The OFF delay in our model, inspired by the findings of Kerschensteiner and Wong (*22*), allows the ON and OFF subfields to segregate and thereby establishes orientation-tuned SC neurons. This temporally delayed mechanism might play a role in establishing different functional features by strengthening different RGC types. For example, the transient and sustained RGC types could organize in a spatiotemporal way such that direction selectivity can emerge from the selective strengthening of these RGC types (*44*). Hence, because of its simplicity, our model can be easily extended to incorporate the establishment of other functional maps, e.g., the motion direction preference map. A straightforward postulation of the extended model would be that the direction preference is in the same direction as, and therefore parallel to, the net local wave vector. Li *et al.* (*10*) have observed that the direction preference in the mouse SC is perpendicular to the orientation preference, which is similar to the angular difference between the net wave vector and the orientation preference in our model (Fig. 4, C_2_ and D_2_), consistent with the postulation. However, de Malmazet *et al.* (*11*) did not observe an obvious relationship between the orientation preference and direction preference maps, and the existence of a direction preference map in the SC is still debatable (*4*, *12*). Kasai and Isa (*45*) recently reported that the brain state (awake or anesthetized) can affect the organization of the functional maps, providing a possible explanation for these inconsistencies in the literature. Given the diversity in the experimental observations, the underlying mechanisms that give rise to the direction preference in the SC might be much more complicated than what we postulated above.

The mechanism used in this model for the emergence of orientation-tuned SC neurons is based on the convergent ON/OFF inputs as in the cortical simple cells. The establishment of the net ON/OFF subfields in the SC neurons requires only slight nonoverlap between the ON and OFF retinal inputs (Fig. 3E), which is consistent with the SC RFs measured in vivo that have large, but not fully, overlapping area between the ON and OFF subfields (*7*). We observed that a subset of SC neurons have the orientation preference that could be estimated from the configuration of their ON and OFF subfields (fig. S1), suggesting that this mechanism is likely to exist in the retinocollicular pathway. Moreover, the SC RFs without the cortical inputs have reduced ON/OFF overlap (*7*), implying that the ON and OFF retinal inputs to the SC are even more segregated than one would measure with cortical feedback. Our developmental model emphasizes the establishment of a concentric functional organization from developmental mechanisms, and we showed the feasibility of ON/OFF convergent inputs in shaping the orientation-tuned SC neurons under this developmental framework. However, we believe that the orientation-tuned SC neurons could arise from multiple mechanisms. Other potential mechanisms for the development of orientation-tuned SC neurons include inheritance of orientation-selective RGCs from the retina (*46*), convergence of opposite direction–selective RGCs (*47*), and elongated RF (*46*, *48*). Since our developmental model postulates that the net retinal wave flow can act as scaffolding for the emergence of OPM, it is conceivable that these other mechanisms are also compatible with our proposed developmental framework.

Although the model can recapitulate several key findings about the SC OPM, e.g., the concentric organization and alignment to the optic flow, there are some limitations of the model. First, we used a simplified probabilistic, rather than detailed mechanistic, approach to model the asymmetric inhibition, which might overlook some key details of the asymmetric inhibition process. Besides, we assumed that the model is only retinotopically mapped from the retina to the SC, and no other functional properties are established at the beginning of the simulations, which ignored the potential that the S2 retinal waves might have already shaped the functional maps to some extent. Nevertheless, it is still reasonable to believe that the simplified mechanisms of our model do contribute considerably to the shaping and maturation of the SC OPM.

Despite its simplicity, the model provides a set of predictions that could be tested in vivo. (i) Interfering with the wave direction bias, but leaving the OFF delay intact, should result in a different SC OPM. In case of uniform wave directions, it should be a concentric OPM with its singularity located at the center, instead of the anterior part, of the SC. This can be tested in vivo by applying gabazine to block the asymmetric GABAergic inhibition by starburst amacrine cells in the retina during development (*13*). (ii) In our model, the OFF receptive subfield is oriented toward the center of the retinal wave flow, which is also the visual field center. This can be validated by mapping the ON and OFF subfields of orientation-selective SC neurons. However, this prediction is based on the assumption that the orientation preference emerges because of the convergence of ON and OFF RGCs. Other potential mechanisms for the emergence of orientation preference in SC neurons, such as inheritance of orientation-selective RGCs from the retina (*46*) or convergence of opposite direction–selective RGCs (*47*), might not comply with this prediction. (iii) In our model, the tuning strength of SC neurons varies systematically across the SC OPM, with strongly tuned neurons being located in regions with large net wave vectors. Our model predicts that the region around the OPM singularity has relatively low tuning strength (Fig. 4, C and D) and is more susceptible to the noise (Fig. 6). This, in turn, provides a plausible alternative explanation for the existing controversy in the literature mentioned above regarding the functional map organization. For example, Wang *et al.* (*7*) and Inayat *et al.* (*4*) recorded in more anterior SC, which might be more susceptible to the activity noise during development that results in a functional organization that is not spatially clustered, whereas Feinberg and Meister (*9*) recorded in more posterior SC, which is more robust to the activity noise (Fig. 6B). (iv) As our model suggests that the main role of the wave direction bias is to align the concentric OPM to the optic flow (Fig. 5), we can deduce that there is relatively less, or even no, retinal wave bias in the animals with binocular vision. This is because the center of the translatory optic flow in the binocular vision is in a more central part of the retina compared to the monocular vision, thus requiring less wave direction bias to shift the OPM to match the optic flow. To the best of our knowledge, there is currently no study reporting the wave direction bias in the developing retina of binocular animals. Therefore, future experimental studies are required to verify this model prediction.

Many theories and mechanisms have been proposed for the establishment of OPM in the V1 of higher mammals (*30*, *31*, *35*, *37*, *49*–*58*). However, the OPM in the SC is less well studied. Because of the difference between the V1 and SC OPMs, where V1 has equal representation for all orientations in every retinotopic location (*59*), whereas SC has retinotopically biased representation of the orientations (*8*, *9*), the proposed mechanisms for the V1 OPM may not be suitable for explaining the SC OPM. Our model helps bridge this gap by showing the viability of retinal waves in shaping the SC OPM. It is well known that retinal waves are key factors for refining the retinotopic map in the visual system, including SC (*16*, *27*, *60*). However, how functional response properties of SC neurons are established during development by the retinal waves remain largely unknown. Our modeling work now suggests that there is a much more direct link between the retinal wave flow statistics and the SC functional maps than previously thought. Thus, spontaneous activity during development not only refines the retinotopic map (*16*) but also has further potentials in establishing the functional organization in the target regions. Together, our model provides a plausible framework for associating developmental properties in developing animals to the functional organization in adult animals. The parsimonious explanations offered by our model outline a promising path toward understanding the design of the functional circuits by the developmental mechanisms, which has been a long-standing question in many different brain regions.

## MATERIALS AND METHODS

### Two-layer retina-SC model

The model has two layers. The first layer models a retina that contains 1600 ON RGCs and 1600 OFF RGCs, each arranged in a 40 × 40 grid. The length of a pixel is 50 μm. The second layer has 1600 SC neurons, also arranged in a 40 × 40 grid. The RGCs were connected to the SC neurons retinotopically (Eq. 14), i.e., the neighboring RGCs projected to similar retinotopic locations in the SC. The synaptic weights were initialized uniformly with strength of 0.1. The diameter of the integration site of RGC inputs, which is the sum of the average RGC axonal diameter and the average dendritic diameter of SC neurons, was set to 350 μm (*21*, *27*, *28*), and its radius *R*_radius_ = 175 μm was used for defining the arbor function (Eq. 14).

The retinal positions are denoted by Greek alphabets $\vec{\alpha },\vec{\beta },\ldots$, and the SC positions are denoted by Roman alphabets $\vec{x},\vec{y},\ldots$, which are all 2D vectors. Since the model assumed that the presynaptic RGCs have already been retinotopically mapped to the postsynaptic SC neurons, the location vectors $\vec{\alpha }$ and $\vec{x}$ also reflect the RF position in the visual field ${\vec{\alpha }}^{\mathrm{VF}}$ (Eq. 20). For all illustrations in this model, the top, right, bottom, and left refer to dorsal (D), temporal (T), ventral (V), and nasal (N) in the visual field, respectively. Consequently, these directions correspond to V, N, D, and T in the retina and medial (M), posterior (P), lateral (L), and anterior (A) in the SC (Fig. 1, A and C) (*13*).

Two functions that were frequently used are the 2D Gaussian function and the polar bias index. The 2D Gaussian function used here was defined as

$$G(\vec{x};{\vec{x}}_{0},\sigma )=\frac{1}{2\pi \sigma^{2}}\exp\left(-\frac{∥\;\vec{x}-{\vec{x}}_{0}{∥}^{2}}{2\sigma^{2}}\right)$$ (1)

where ∥. ∥ denotes the Euclidean norm, ${\vec{x}}_{0}$ is the center, and σ is the spread.

The polar bias index, *B*, was computed by

$$B=\frac{|\int_{-\pi }^{\pi }p(\theta )\exp(i\theta )d\theta |}{\int_{-\pi }^{\pi }p(\theta )d\theta }$$ (2)

where *p* is a polar probability density function truncated for the interval (−π, π] and *p*(θ) is the probability for a direction θ ∈ (−π, π]. Since the normalization term $\int_{-\pi }^{\pi }p(\theta )d\theta =1$, *B* is reduced to

$$B=|\int_{-\pi }^{\pi }p(\theta )\exp(i\theta )d\theta |$$ (3)

### Retinal wave model

Each wave *w* was initiated at position ${\vec{\alpha }}_{w}^{\mathrm{init}}$ one after another by sampling from the uniformly distributed wave initiation map (*13*). The temporal resolution *T*_frame_, i.e., the duration per frame of wave, was set to 0.5 s. To implement the asymmetric inhibition shown in (*13*), a source position of the asymmetric inhibition ${\vec{\alpha }}_{w}^{\mathrm{AI}}$ was sampled from a 2D Gaussian distribution centered at the putative nose position (center of vision) ${\vec{\alpha }}^{\mathrm{nose}}=\left[\begin{array}{c} \alpha_{\mathrm{AP}}^{\mathrm{nose}}\\ \alpha_{\mathrm{ML}}^{\mathrm{nose}} \end{array}\right]=\left[\begin{array}{c} 250\\ 1000 \end{array}\right]$ μm with spread σ^AI^ ∈ {5,25,50,150,250,350,500,600,750,1000,1500,2000,3000} μm (Fig. 5).

### Wave propagation

The activation state of the RGC at the position $\vec{\alpha }$ in the wave frame *t* was described by

$$A(\vec{\alpha },t)=\left\{ \begin{array}{cc} 1, & \mathrm{if}\;N_{\vec{\alpha }}^{\mathrm{active}}(t-1)\geq N_{\vartheta }\;\mathrm{and}\;t>t_{\vec{\alpha }}^{\prime }+\tau_{\mathrm{IEI}}\\ 0, & \mathrm{otherwise} \end{array}\right.$$ (4)

where $N_{\vec{\alpha }}^{\mathrm{active}}(t)=\sum_{\vec{\beta }}X_{\rho_{\vec{\beta }}}$ is the total number of successful activations by active $\vec{\beta }$ nearest neighbors (NNs) of the $\vec{\alpha }$ RGC at time *t* with $X_{\rho_{\vec{\beta }}}∼B(1,\rho_{\vec{\beta }})$ as the sample from a binomial distribution with the activation probability $\rho_{\vec{\beta }}$, *N*_ϑ_ = 1 is the threshold number of successful activations by the active $\vec{\beta }$ NNs, $t_{\vec{\alpha }}^{\prime }$ is the time of last activation for the RGC at $\vec{\alpha }$, and τ_IEI_ = 60 frames = 30 s is the inter-event interval (IEI) (*14*).

The local area for position $\vec{\alpha }$, $a_{\vec{\alpha }}$, was defined as the 3 × 3 pixels centered at $\vec{\alpha }$. To induce directed waves, the activation probability $\rho_{\vec{\beta }}$ for the $\vec{\beta }$ NNs of an active $\vec{\alpha }$ RGC was not uniformly distributed and was determined by the polar probability density function for local propagation *p*^prop^

$$\frac{\rho_{\vec{\beta }}}{q}=p^{\mathrm{prop}}(\theta_{\vec{\beta }};\sigma^{\mathrm{prop}})=\frac{g(\theta_{\vec{\beta }};\sigma^{\mathrm{prop}})}{\sum_{\vec{\gamma }}g(\theta_{\vec{\gamma }};\sigma^{\mathrm{prop}})};\theta_{\vec{\beta }},\theta_{\vec{\gamma }}\in (-\pi ,\pi ];\vec{\beta },\vec{\gamma }\in a_{\vec{\alpha }};\vec{\beta },\vec{\gamma }\neq \vec{\alpha }$$ (5)

where *q* = 2.4 is a scaling factor and

$$g(\theta ;\sigma )=\frac{1}{\sigma \sqrt{2\pi }}\exp\left(-\frac{\theta^{2}}{2\sigma^{2}}\right)$$ (6)

is the 1D Gaussian function truncated for the interval (−π, π] with mean of 0 and SD σ. The SD σ^prop^ was numerically optimized by

$$\sigma^{\mathrm{prop}}=\arg\;\min_{\sigma \in R^{+}}{\left[b-B(\sigma )\right]}^{2}$$ (7)

where *b* = 0.35 is the desired local propagation bias and

$$B(\sigma )=|\;\sum {{}_{\vec{\beta }\in a_{\vec{\alpha }},\vec{\beta }\neq \vec{\alpha }}p}^{\mathrm{prop}}(\theta_{\vec{\beta }};\sigma )\exp(i\theta_{\vec{\beta }})|$$ (8)

is the discrete version of Eq. 3 for calculating the bias of the local propagation probability density function *p*^prop^. $\theta_{\vec{\beta }}=\arg({\vec{\nu }}_{x}+i{\vec{\nu }}_{y})$, where $\vec{\nu }=\vec{\beta }-\vec{\alpha }$. In the square grid configuration of the model, $\theta_{\vec{\beta }}$ can also be expressed as θ*_k_*, where $\theta_{k}=k\frac{2\pi }{N}$ is the direction of *k*th NN at $\vec{\beta }$ relative to the RGC at $\vec{\alpha }$ and *N* = 8 is the total number of NNs. The *b* controls the shape and size of the retinal wave, where higher *b* will produce smaller and straighter waves that resemble the S3 waves, and lower *b* will produce larger, curved waves that resemble the S2 waves (fig. S2). However, in this model, the main effect of the wave size is the coverage on the SC. A large wave coverage requires less number of waves to develop the orientation map. Figure S7 shows an example of the development of an orientation map over the wave iterations (wave coverage) using *b* = 0.55. The wave propagation direction was computed as $\theta_{w}^{\mathrm{wave}}=\arg({\vec{\mu }}_{x}^{w}+i{\vec{\mu }}_{y}^{w})$, where ${\vec{\mu }}^{w}={\vec{\alpha }}_{w}^{\mathrm{init}}-{\vec{\alpha }}_{w}^{\mathrm{AI}}$. Next, $\theta_{w}^{\mathrm{wave}}$ was rounded to the nearest θ*_k_* to get $\theta_{w}^{\mathrm{wave}\prime }=\frac{2\pi }{N}\left\lfloor \frac{N\theta_{w}^{\mathrm{wave}}}{2\pi }+0.5\right\rfloor$. The peak probability of *p*^prop^ was then oriented to $\theta_{w}^{\mathrm{wave}\prime }$ such that $\rho_{\vec{\beta }}=p^{\mathrm{prop}}(\theta_{\vec{\beta }}-\theta_{w}^{\mathrm{wave}\prime };\sigma^{\mathrm{prop}})$. In other words, the wave *w* was propagating from its initiation position ${\vec{\alpha }}_{w}^{\mathrm{init}}$ away from the source position of the asymmetric inhibition ${\vec{\alpha }}_{w}^{\mathrm{AI}}$ (fig. S2).

### RGC activity

The activity of the active RGCs was modeled as firing rate represented by a response kernel defined by

$$K(t)=r\chi_{\left(0,T_{\mathrm{wave}}\right]}(t)+\gamma \xi (t)$$ (9)

where *r* is the mean RGC firing rate, *T*_wave_ = 1 s is the pixel activation duration during the wave (*14*), γ is the strength for noise and was set to 0 if not stated otherwise, ξ(*t*) is the Gaussian white noise, and χ*_T_*(*t*) is the indicator function for interval *T*

$$\chi_{T}(t)=\left\{ \begin{array}{cc} 1, & \mathrm{if}\;t\in T\\ 0, & \mathrm{if}\;t\notin T \end{array}\right.$$ (10)

The response kernel for RGCs with polarity *P* and delay τ*_P_* was given by

$$K_{P}(t)=K(t-\tau_{P}),P\in \{\mathrm{ON},\mathrm{OFF}\}$$ (11)

τ_ON_ = 0 s and τ_OFF_ = 1 s were used in the simulations to mimic the delayed OFF activation (*22*) unless stated otherwise.

The RGC activity of polarity *P* at location $\vec{\alpha }$ at time *t* was computed by

$$V_{P}^{\mathrm{Pre}}(\vec{\alpha },t)=\sum_{t^{\prime }=1}^{t}A(\vec{\alpha },t^{\prime })K_{P}(t-t^{\prime })$$ (12)

This equation implies that the activity of OFF RGCs is just a copy of the ON-RGC activity with a delay of τ_OFF_ − τ_ON_.

### Learning mechanism

Since the activity of postsynaptic SC neurons is highly correlated to the presynaptic stage III RGC activity (*14*), Hebbian learning rule (*35*, *36*) with subtractive normalization (*37*, *38*, *61*–*64*) was used to update the synaptic weights between the presynaptic RGCs and the postsynaptic SC neurons. The postsynaptic activity of SC neuron at location $\vec{x}$ is a weighted sum of all its presynaptic inputs $V_{P}^{\mathrm{Pre}}(\vec{\alpha },t)$

$$V^{\mathrm{Post}}(\vec{x},t)=\sum_{\vec{\alpha },P}w_{P}(\vec{x},\vec{\alpha },t)V_{P}^{\mathrm{Pre}}(\vec{\alpha },t)$$ (13)

where $w_{P}(\vec{x},\vec{\alpha },t)=J_{P}(\vec{x},\vec{\alpha },t)R(\vec{x},\vec{\alpha })$ is the synaptic weight at time *t* from RGC of polarity *P* at $\vec{\alpha }$ to SC neuron at $\vec{x}$. $J_{P}(\vec{x},\vec{\alpha },t)$ gives the synaptic weight of all presynaptic-postsynaptic pairs, and

$$R(\vec{x},\vec{\alpha })=\left\{ \begin{array}{cc} 1, & \mathrm{if}∥\;\vec{x}-\vec{\alpha }\;∥\leq R_{\mathrm{radius}}\\ 0, & \mathrm{otherwise} \end{array}\right.$$ (14)

is the arbor function (input integration site) for ensuring that the influence is constrained by the retinotopy. *R*_radius_ = 175 μm is two times the radius of the axonal arbor estimated from (*21*, *27*, *28*).

The initial synaptic weight *w*_0_ was set to 0.1. The synaptic weight update was computed by

$$\frac{d}{dt}w_{P}(\vec{x},\vec{\alpha },t)=\eta V^{\mathrm{Post}}(\vec{x},t)[V_{P}^{\mathrm{Pre}}(\vec{\alpha },t)-{\left\langle V^{\mathrm{pre}}(t)\right\rangle }_{\vec{x}}]$$ (15)

where η is the learning rate and

$${\left\langle V^{\mathrm{pre}}(t)\right\rangle }_{\vec{x}}=\frac{V^{\mathrm{Post}}(\vec{x},t)}{2\sum_{\vec{\alpha }}R(\vec{x},\vec{\alpha })}$$ (16)

is the weighted average activity of the presynaptic RGCs that have projection to the postsynaptic SC neuron at location $\vec{x}$. To speed up the computation, we binned the time *t* into trials *T_i_* = *iT*_trial_ and updated the synaptic weights after each trial by averaging over the presynaptic activities. By substituting Eqs. 13 and 16 into Eq. 15, we have

$$\begin{array}{c} \frac{d}{dT_{i}}w_{P}(\vec{x},\vec{\alpha },T_{i})=\eta \sum_{\vec{\beta },P{}^{\prime }}w_{P{}^{\prime }}(\vec{x},\vec{\beta },T_{i})V_{P^{\prime }}^{\mathrm{Pre}}(\vec{\beta },T_{i})\left[V_{P}^{\mathrm{Pre}}(\vec{\alpha },T_{i})-\frac{\sum_{\vec{\gamma },P^{\prime \prime }}w_{P^{\prime \prime }}(\vec{x},\vec{\gamma },T_{i})V_{P^{\prime \prime }}^{\mathrm{Pre}}(\vec{\gamma },T_{i})}{2\sum_{\vec{\gamma }}R(\vec{x},\vec{\gamma })}\right]\\ =\eta \left[\sum_{\vec{\beta },P^{\prime }}w_{P^{\prime }}(\vec{x},\vec{\beta },T_{i})C^{P,P^{\prime }}\left(\vec{\alpha },\vec{\beta },T_{i}\right)-\frac{\sum_{\vec{\beta },\vec{\gamma },P^{\prime },P^{\prime \prime }}w_{P^{\prime }}(\vec{x},\vec{\beta },T_{i})w_{P^{\prime \prime }}(\vec{x},\vec{\gamma },T_{i})C^{P^{\prime },P^{\prime \prime }}(\vec{\beta },\vec{\gamma },T_{i})}{2\sum_{\vec{\gamma }}R(\vec{x},\vec{\gamma })}\right] \end{array}$$ (17)

where

$$C^{P,P^{\prime }}(\vec{\alpha },\vec{\beta },T_{i})=\frac{1}{T_{\mathrm{trial}}}\sum_{t=T_{i-1}+1}^{T_{i}}V_{P}^{\mathrm{Pre}}(\vec{\alpha },t)V_{P\prime }^{\mathrm{Pre}}(\vec{\beta },t)$$ (18)

*T*_trial_ is the trial duration in frames and *i* ∈ ℤ^+^. To prevent the synaptic weights from becoming negative, the synaptic weights were constrained by

$$w_{P}(\vec{x},\vec{\alpha },t)=\left\{ \begin{array}{cc} w_{P}(\vec{x},\vec{\alpha },t)-\frac{w_{P}(\vec{x},\vec{\alpha },t)\sum_{\vec{\beta },P}w_{P}^{-}(\vec{x},\vec{\beta },t)}{\sum_{\vec{\beta },P}w_{P}^{+}(\vec{x},\vec{\beta },t)}, & \mathrm{if}\;w_{P}(\vec{x},\vec{\alpha },t)>0\\ \;0, & \mathrm{otherwise} \end{array}\right.$$ (19)

after updating the synaptic weights of each trial. $w_{P}^{-}(\vec{x},\vec{\beta },t)$ gives the absolute value of the negative weights, and $w_{P}^{+}(\vec{x},\vec{\beta },t)$ gives the positive weights from the *P* RGC at $\vec{\beta }$ to the postsynaptic SC neuron at $\vec{x}$. Together, the subtractive normalization (Eq. 15) and the weight constraint (Eq. 19) ensured that the total synaptic weight between all RGCs and each SC neuron is conserved, i.e., $\sum_{\vec{\alpha },P}\frac{d}{dt}w_{P}(\vec{x},\vec{\alpha },t)=0$, while keeping all synaptic weights nonnegative. These mechanisms were used to mimic the response homeostasis (*65*).

### Receptive field

The RF of RGC at location $\vec{\alpha }$ with polarity *P*, ${\text{Ψ}}_{P,\vec{\alpha }}^{\mathrm{RGC}}$, was modeled as 2D difference of Gaussian in the visual field coordinates ${\vec{\alpha }}^{\mathrm{VF}}=m\vec{\alpha }$, with *m* = 2.5 as the scaling factor from retinal to visual field coordinates

$${\text{Ψ}}_{P,\vec{\alpha }}^{\mathrm{RGC}}={\text{Ψ}}_{P}^{\mathrm{RGC}}({\vec{\beta }}^{\mathrm{VF}};{\vec{\alpha }}^{\mathrm{VF}},\sigma^{\mathrm{RF}})=h\left[G({\vec{\beta }}^{\mathrm{VF}};{\vec{\alpha }}^{\mathrm{VF}},\sigma^{\mathrm{RF}})-\frac{1}{5}G({\vec{\beta }}^{\mathrm{VF}};{\vec{\alpha }}^{\mathrm{VF}},3\sigma^{\mathrm{RF}})\right]$$ (20)

where

$$h=\left\{ \begin{array}{cc} 1, & \mathrm{if}\;P=\mathrm{ON}\\ -1, & \mathrm{if}\;P=\mathrm{OFF} \end{array}\right.$$ (21)

σ^RF^ = 17 indicates the width of the RGC RF in visual field pixels. The scale is set to 0.6° per visual field pixel.

The RF of the SC neuron at location $\vec{x}$ is the weighted sum of all its presynaptic RGC RFs at time *t*

$${\text{Ψ}}_{\vec{x}}^{\mathrm{SC}}={\text{Ψ}}^{\mathrm{SC}}({\vec{\beta }}^{\mathrm{VF}};\vec{x},t)=\sum_{\vec{\alpha },P}w_{P}(\vec{x},\vec{\alpha },t){\text{Ψ}}_{P,\vec{\alpha }}^{\mathrm{RGC}}$$ (22)

The contrast of the SC RF, ${\text{Ψ}}_{\mathrm{diff}}^{\mathrm{SC}}=\max{\text{Ψ}}^{\mathrm{SC}}-\min{\text{Ψ}}^{\mathrm{SC}}$, is the difference between its maximal and minimal values.

### Analyses

#### Net wave vector

The local net wave vector of location $\vec{\alpha }$ over its local area $a_{\vec{\alpha }}$ (area of 3 × 3 pixels centered at $\vec{\alpha }$) was computed by

$${\vec{u}}_{\vec{a}}=\sum_{t}\vec{v}(\vec{a},t)$$ (23)

where

$$\vec{\nu }(\vec{\alpha },t)=\vec{c}(a_{\vec{\alpha }},t)-\vec{c}(a_{\vec{\alpha }},t-1)$$ (24)

is the instantaneous wave vector at time *t* and

$$\vec{c}(a_{\vec{\alpha }},t)=\frac{\sum_{\vec{\beta }\in a_{\vec{\alpha }}}A(\vec{\beta },t)\vec{\beta }}{\sum_{\vec{\alpha }\in a_{\vec{\alpha }}}A(\vec{\beta },t)}$$ (25)

is the center of mass for the activity within the area $a_{\vec{\alpha }}$ at time *t*. The magnitude of the wave vector is $∥\;{\vec{u}}_{\vec{\alpha }}\;∥$, and the direction is $\arg({\vec{u}}_{\vec{\alpha }x}+i{\vec{u}}_{\vec{\alpha }y})$.

#### Wave propagation direction bias

The bias of the wave propagation direction, *B*^wave^, was computed by

$$B^{\mathrm{wave}}=|\int p^{\mathrm{wave}}(\theta^{\mathrm{wave}})\exp(i\theta^{\mathrm{wave}})d\theta^{\mathrm{wave}}|$$ (26)

where *p*^wave^ is the normalized frequency distribution of the wave propagation directions θ^wave^.

#### Orientation map analyses

The orientation preference θ_pref_ = arg (μ)/2 of the SC neuron was estimated from the Fourier transform of its RF (*30*), ${\hat{\text{Ψ}}}^{\mathrm{SC}}(\vec{\omega })$, where

$$\mu =\frac{\int |{\hat{\text{Ψ}}}^{\mathrm{SC}}(\vec{\omega })|∥\;\vec{\omega }\;∥\exp[2i\arg(\vec{\omega })]d\vec{\omega }}{\int |{\hat{\text{Ψ}}}^{\mathrm{SC}}(\vec{\omega })|d\vec{\omega }}$$ (27)

and $\vec{\omega }$ is the vector of spatial frequency components. gOSI was computed by

$$I^{\mathrm{gOS}}=\frac{|\int L(\theta )\exp(2i\theta )d\theta |}{\int L(\theta )d\theta }$$ (28)

where

$$L(\theta )=\int |{\hat{\text{Ψ}}}^{\mathrm{SC}}({\vec{\omega }}_{\theta })|d{\vec{\omega }}_{\theta }$$ (29)

is the orientation tuning curve (Fig. 3C) obtained by summing over the frequencies ${\vec{\omega }}_{\theta }$ for a given orientation $\theta =\arg({\vec{\omega }}_{\theta })$. The tuning strength *L*_max_ = max *L*(θ) is the maximal value of the tuning curve *L*(θ). The singularity of the concentric OPM was estimated from the minimal wave flow magnitude.

#### ON/OFF segregation index

The segregation of the ON and OFF inputs to SC neuron at location $\vec{x}$ at time *t* was computed by

$$I^{\mathrm{SEG}}(\vec{x},t)=S^{\mathrm{ratio}}(\vec{x},t)S^{\mathrm{amount}}(\vec{x},t)$$ (30)

where

$$\begin{array}{rl} S^{\mathrm{ratio}}(\vec{x},t) & =1-\frac{|\;\sum_{\vec{\alpha }}W^{D+}(\vec{x},\vec{\alpha },t)-\sum_{\vec{\alpha }}W^{D-}(\vec{x},\vec{\alpha },t)|}{\sum_{\vec{\alpha }}W^{D+}(\vec{x},\vec{\alpha },t)+\sum_{\vec{\alpha }}W^{D-}(\vec{x},\vec{\alpha },t)}\\ & =1-\frac{|\;\sum_{\vec{\alpha }}W^{D}(\vec{x},\vec{\alpha },t)|}{\sum_{\vec{\alpha }}|W^{D}(\vec{x},\vec{\alpha },t)|} \end{array}$$ (31)

measures how balanced the ON and OFF inputs are [perfectly balanced means ON-to-OFF ratio at location $\vec{x}$ is 1, and thus, $S^{\mathrm{ratio}}(\vec{x},t)=1$], i.e., the inverse of the overall net ON or OFF input to the postsynaptic SC neuron at location $\vec{x}$ (the inverse of absolute ON/OFF dominance), and

$$S^{\mathrm{amount}}(\vec{x},t)=\frac{\sum_{\vec{\alpha }}|W^{D}(\vec{x},\vec{\alpha },t)|}{\sum_{\vec{\alpha }}W^{S}(\vec{x},\vec{\alpha },t)}$$ (32)

measures the amount of net ON and OFF inputs from each $\vec{\alpha }$ in comparison to total inputs, which is the amount of nonoverlapping ON and OFF inputs to the postsynaptic SC neuron at location $\vec{x}$. $S^{\mathrm{amount}}(\vec{x},t)$ is equivalent to DSEG (degree of segregation) in (*36*), and the nonabsolute version of $S^{\mathrm{amount}}(\vec{x},t)$ is equivalent to SIGN in (*36*) and segregation index in (*34*). $W^{D}(\vec{x},\vec{\alpha },t)=w^{\mathrm{ON}}(\vec{x},\vec{\alpha },t)-w^{\mathrm{OFF}}(\vec{x},\vec{\alpha },t)$ gives the net ON (positive) or OFF (negative) input from location $\vec{\alpha }$ to the SC neuron at location $\vec{x}$, and $W^{S}(\vec{x},\vec{\alpha },t)=w^{\mathrm{ON}}(\vec{x},\vec{\alpha },t)+w^{\mathrm{OFF}}(\vec{x},\vec{\alpha },t)$ gives the total ON and OFF inputs from location $\vec{\alpha }$ to the SC neuron at location $\vec{x}$. By substituting Eqs. 31 and 32 into Eq. 30, we have

$$I^{\mathrm{SEG}}(\vec{x},t)=\frac{\sum_{\vec{\alpha }}|W^{D}(\vec{x},\vec{\alpha },t)|-|\sum_{\vec{\alpha }}W^{D}(\vec{x},\vec{\alpha },t)|}{\sum_{\vec{\alpha }}W^{S}(\vec{x},\vec{\alpha },t)}$$ (33)

Both $S^{\mathrm{ratio}}(\vec{x},t)$ and $S^{\mathrm{amount}}(\vec{x},t)$ have values in the range [0,1]; therefore, $I^{\mathrm{SEG}}(\vec{x},t)\in [0,1]$. Similarly, the same formulation as Eq. 33 can be used to compute the ON/OFF subfield segregation $I_{\text{Ψ}}^{\mathrm{SEG}}$ (fig. S4B) between the *P* subfields of the SC RF ${\text{Ψ}}_{\vec{x},P}^{\mathrm{SC}}={\text{Ψ}}_{P}^{\mathrm{SC}}({\vec{\alpha }}^{\mathrm{VF}};\vec{x},t)=\sum_{\vec{\beta }}w_{P}(\vec{x},\vec{\beta },t){\text{Ψ}}_{P,\vec{\beta }}^{\mathrm{RGC}}$ by replacing $W^{D}(\vec{x},\vec{\alpha },t)$ with ${\text{Ψ}}^{D}({\vec{\alpha }}^{\mathrm{VF}};\vec{x},t)={\text{Ψ}}_{\vec{x},\mathrm{ON}}^{\mathrm{SC}+}-{\text{Ψ}}_{\vec{x},\mathrm{OFF}}^{\mathrm{SC}-}$ and $W^{S}(\vec{x},\vec{\alpha },t)$ with ${\text{Ψ}}^{S}({\vec{\alpha }}^{\mathrm{VF}};\vec{x},t)={\text{Ψ}}_{\vec{x},\mathrm{ON}}^{\mathrm{SC}+}+{\text{Ψ}}_{\vec{x},\mathrm{OFF}}^{\mathrm{SC}-}$

$$I_{\text{Ψ}}^{\mathrm{SEG}}(\vec{x},t)=\frac{\sum_{{\vec{\alpha }}^{\mathrm{VF}}}|{\text{Ψ}}^{D}({\vec{\alpha }}^{\mathrm{VF}};\vec{x},t)|-|\sum_{{\vec{\alpha }}^{\mathrm{VF}}}{\text{Ψ}}^{D}({\vec{\alpha }}^{\mathrm{VF}};\vec{x},t)|}{\sum_{{\vec{\alpha }}^{\mathrm{VF}}}{\text{Ψ}}^{S}({\vec{\alpha }}^{\mathrm{VF}};\vec{x},t)}$$ (34)

where ${\text{Ψ}}_{\vec{x},\mathrm{ON}}^{\mathrm{SC}+}$ denotes the positive part of the ON subfield and ${\text{Ψ}}_{\vec{x},\mathrm{OFF}}^{\mathrm{SC}-}$ denotes the absolute value of the negative part of the OFF subfield.

#### Local homogeneity index

LHI is a measure of how similar the local orientation preferences are (*39*). LHI for location $\vec{x}$ was computed by

$$I^{\mathrm{LH}}(\vec{x})=|\int G(\vec{y};\vec{x},\sigma^{\mathrm{LH}})\exp(i2\theta_{\vec{y}})d\vec{y}|$$ (35)

where $\theta_{\vec{y}}$ is the orientation preference at location $\vec{y}$ and σ^LH^ = 100 μm.

### In vivo experiments

#### Animals, surgery, and preparation

All experimental procedures were performed in accordance with the guidelines of the local authority (LAGeSo Berlin, G0142/18). Maximal care was given to minimize the number and discomfort of used animals. C57BL/6J adult male mice were bred locally (Charité-Forschungseinrichtung für Experimentelle Medizin; *n* = 13). Induction, surgery, and recording were done under isoflurane (2.5% in oxygen, Cp-Pharma, G227L19A). Methods, surgery, and animal exposure were performed as previously reported (*24*, *25*).

#### Electrophysiological recordings and spike sorting

Neuronal activity in the mouse SC was recorded with Neuropixels probes using the Open Ephys software (www.open-ephys.org). The SC was stereotactically targeted using a tangential insertion angle (*24*, *25*). The extracellular neuronal signals were spike-sorted using Kilosort (Kilosort 2 and 2.5). Manual curation using Phy2 (https://github.com/cortex-lab/phy) was performed on the output from Kilosort. Quality metrics, removal of bad clusters, and controls for double counted spikes were done after the manual curation and before any further analysis. Please refer to (*24*, *25*) for more information regarding the in vivo experiments.

#### RF and orientation tuning analysis

To characterize the RFs, we presented light and dark sparse-noise stimuli. To estimate the RFs, we integrated the visually evoked neuronal activity (integration window, 50 ms) at different latencies (0 to 200 ms; step size, 10 ms) relative to the onset of the sparse-noise targets, resulting in spatiotemporal RFs for both lights and darks (fig. S1A). The ON (light) and OFF (dark) RFs were interpolated by a factor of 2 using a 2D cubic interpolation (scipy.interpolate.interp2d) and normalized in amplitude by the maximum amplitude across all spatiotemporal RFs. The RF was calculated as the pixel-wise maximum of either the ON or OFF RF for each time frame separately. The spatiotemporal RF at the peak responses (±1 frame around the maximum response) was used as the RF of the recorded SC neuron. We only included RFs with high signal-to-noise ratio (SNR ≥6). Moreover, we calculated the number of receptive subfields using skimage.measure.regionprops function. The RFs with more than five subfields were considered as noise and excluded.

To estimate the orientation preference, we Fourier-transformed the RF at the peak response (fig. S1A, right) and extracted the tuning curves (Eq. 29 and fig. S1B). RFs in which the tuning curve extracted from the Fourier transform had an SNR >1.5 were included. To measure the orientation preference in vivo, we presented moving gratings for 8 or 12 directions with temporal frequencies of 2 or 3 Hz and spatial frequencies of 0.02, 0.04, or 0.08 cycles per degree. We quantified the response to the grating stimuli by computing the peristimulus time histogram for each presented direction. The preferred orientation was either extracted by fitting von Mises functions to the orientation tuning curves (fig. S1) or computed by θ_pref_ = arg (μ)/2 (Fig. 1B) where μ = ∑_θ_ ‍ *R*_θ_ exp (2*i*θ)/∑_θ_ ‍ *R*_θ_, where θ is the stimulus direction in radians and *R*_θ_ is the corresponding neuronal response. For fitting the von Mises functions, the tuning curves obtained from the moving-grating responses and estimated from the RFs (fig. S1A) were first interpolated and then fit using the least-squares optimization function from SciPy. The von Mises function is a circular normal distribution, and the sum of two von Mises functions allows fitting direction and orientation tuning curves and extracting preferred orientation. From this fit, we extracted the firing rate of the preferred direction (*R*_pref_), opposite direction (*R*_opp_), and orthogonal direction (*R*_orth_) to calculate the direction selectivity index [*I*^DS^ = (*R*_pref_ − *R*_opp_)/(*R*_pref_ + *R*_opp_)] and orientation selectivity index [*I*^OS^ = (*R*_pref_ − *R*_orth_)/(*R*_pref_ + *R*_orth_)]. In Fig. 1B, only SC neurons with a clear RF and *I*^OS^ > 0.2 were included. The RF position was computed by the center of mass of pixels of the RF Ψ that are above a threshold ϑ_Ψ_ = μ_Ψ_ + 2σ_Ψ_, where Ψ is the sum of absolute value of the ON and OFF RFs, μ_Ψ_ is the mean of Ψ, and σ_Ψ_ is the SD of Ψ. For fig. S1, only SC neurons with a high SNR in the visually evoked activity in response to the moving gratings (SNR >2), good *R*^2^ values for the fits (*R*^2^ > 0.6), *I*^OS^ > 0.5, and *I*^DS^ < 0.3 were considered as orientation-selective neurons (fig. S1, E and F).

## Notes

View/request a protocol for this paper from *Bio-protocol*.
