# Supplementary material for: Retinal waves align the concentric orientation map in mouse superior colliculus to the center of vision
Source: Sci Adv. 2023 May 12;9(19):eadf4240. doi: 10.1126/sciadv.adf4240 (PMC10181181; doi:10.1126/sciadv.adf4240)
Supplement: Supplementary file 1 — Figs. S1 to S9 Legend for data S1 [file sciadv.adf4240_sm.pdf]

Science Advances
AAAS

Supplementary Materials for

Retinal waves align the concentric orientation map in mouse superior colliculus to the center of vision

Kai Lun Teh *et al.*

Corresponding author: Jens Kremkow, jens.kremkow@charite.de

Sci. Adv. **9**, eadf4240 (2023)
DOI: 10.1126/sciadv.adf4240

The PDF file includes:

Figs. S1 to S9
Legend for data S1

Other Supplementary Material for this manuscript includes the following:

Data S1

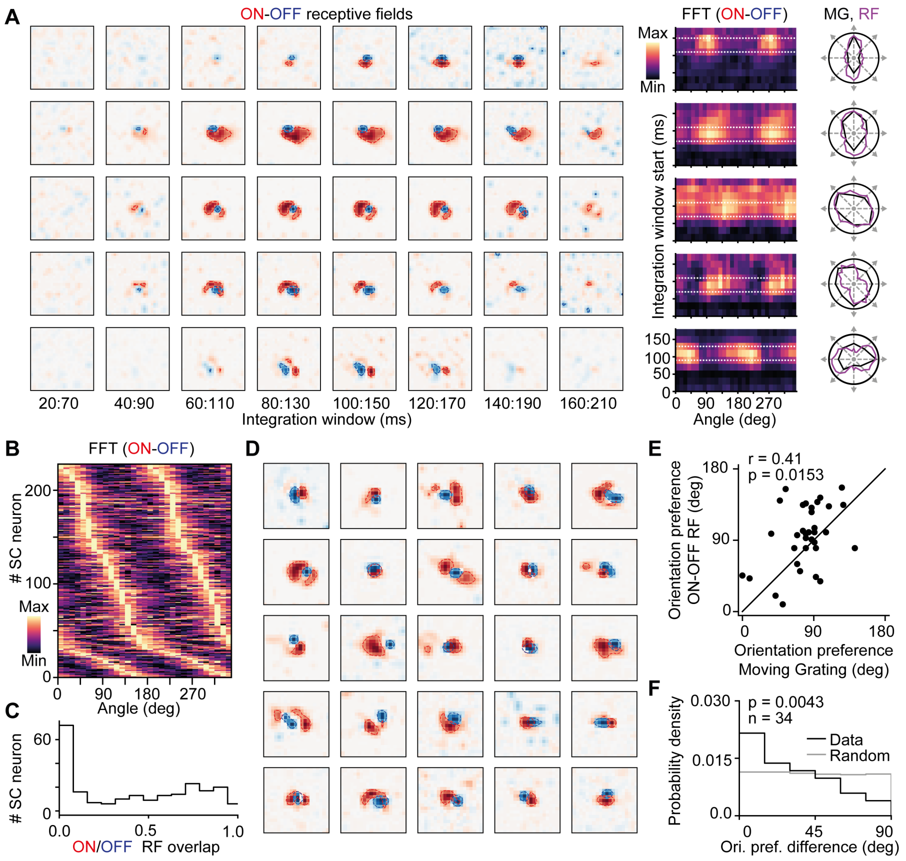

Figure S1: ON/OFF receptive field structure and orientation tuning of SC neurons *in vivo*. (A) Left: spatiotemporal receptive fields of five example SC neurons. The responses to light sparse-noise (ON, red contour lines) and dark sparse-noise stimuli (OFF, blue contour lines) were obtained by integrating (50 ms integration window) the evoked activity at different latencies relative to the stimulus onsets. The color maps show the pixel-wise maximum of the ON/OFF receptive fields. Middle: orientation tuning curves estimated from the receptive fields at different response latencies. Right: polar plot showing the orientation tuning curves obtained from the responses to moving gratings (MG, black) and estimated from the receptive fields (RF, purple). (B) Estimated orientation tuning curves from the receptive field of SC neurons sorted by the predicted preferred orientation ($n = 229$ neurons, SNR > 1.5). (C) Histogram of the overlap of the ON and OFF receptive subfields of the neurons shown in B. It is evident that the ON-OFF overlap varies. (D) Examples of SC neurons with ON/OFF receptive field structure that resembles the receptive fields of SC neurons in the model. As in A, the contour lines show the ON and OFF subfields. A diversity of ON-OFF overlap is evident. (E) Orientation preference measured with moving gratings vs. predicted from the receptive fields of orientation-tuned SC neurons. Pearson correlation coefficient test, $n = 34$ neurons. (F) Absolute difference in the orientation preference measured with moving gratings vs. predicted from the receptive fields for neurons shown in E. A uniform random distribution (min $= 0°$, max $= 90°$, $n = 340$) was used for statistical comparison (Wilcoxon rank-sum test).

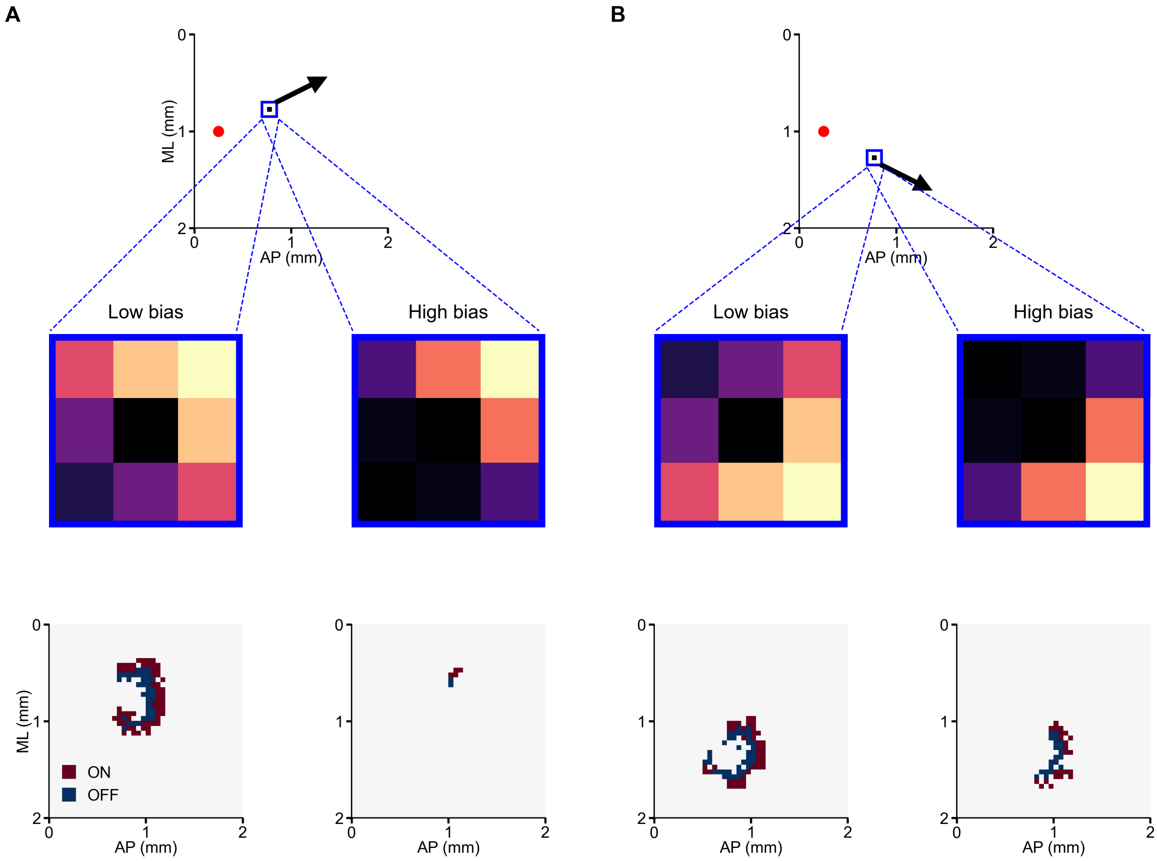

Figure S2: Mechanism for local propagation of the wave. (A) Top: a wave initiation (black pixel) will produce a wave that propagates away from the source of asymmetric inhibition (red dot). Middle: the activation probabilities for the eight nearest neighbors of an activated RGC. A lower local propagation bias (0.35) gives a more evenly distributed activation probabilities of the nearest neighbors (left) whereas a higher local propagation bias (0.65) gives a more focused activation probabilities (right). Bottom: low local propagation bias produces waves that are larger and more curved (left) whereas high local propagation bias produces waves that are smaller and straighter (right). (B) Same as A but for a wave that propagates towards the posterior-lateral direction.

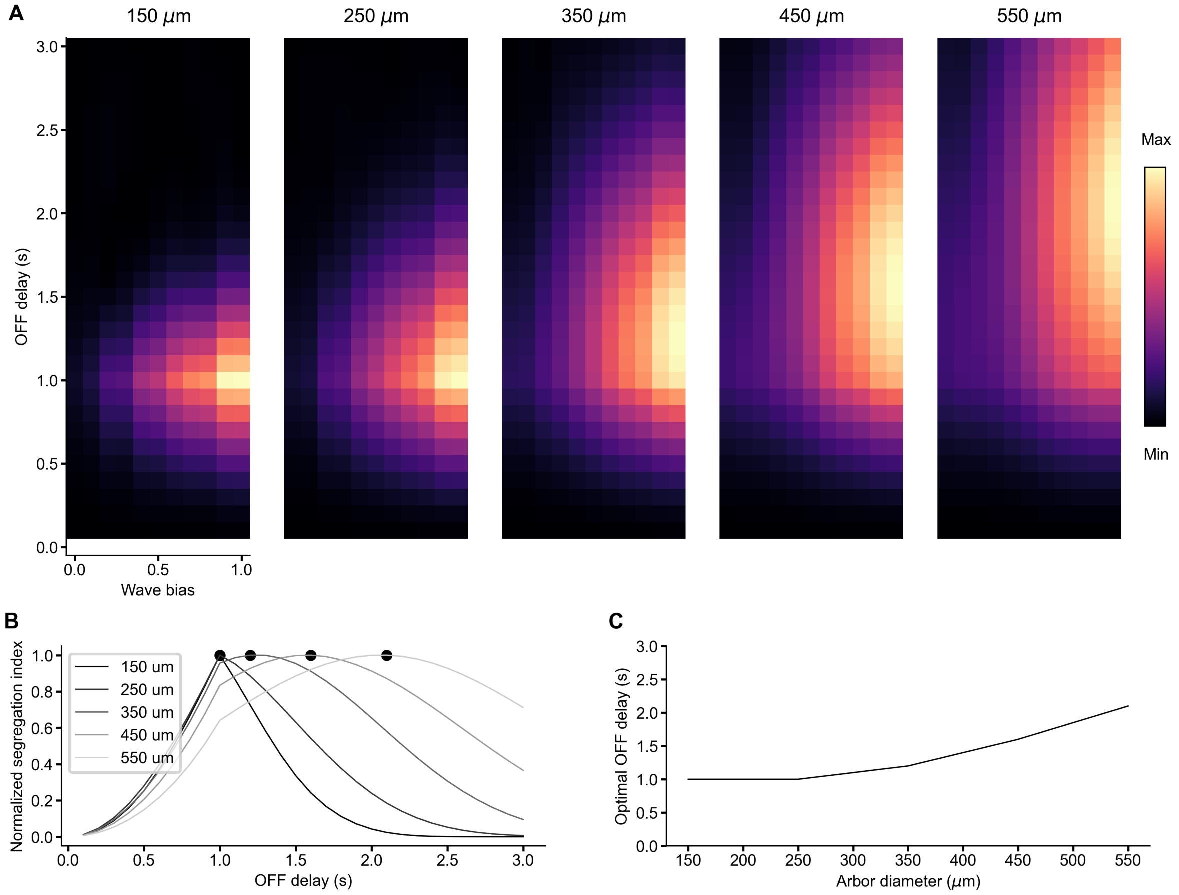

Figure S3: Larger arbor diameter gives larger optimal OFF delay. (A) The ON-OFF input segregation index for different arbor diameters. (B) Normalized ON-OFF input segregation for different arbor diameters with wave direction bias of 1. (C) The optimal OFF delay shifts to larger values as the arbor diameter increases.

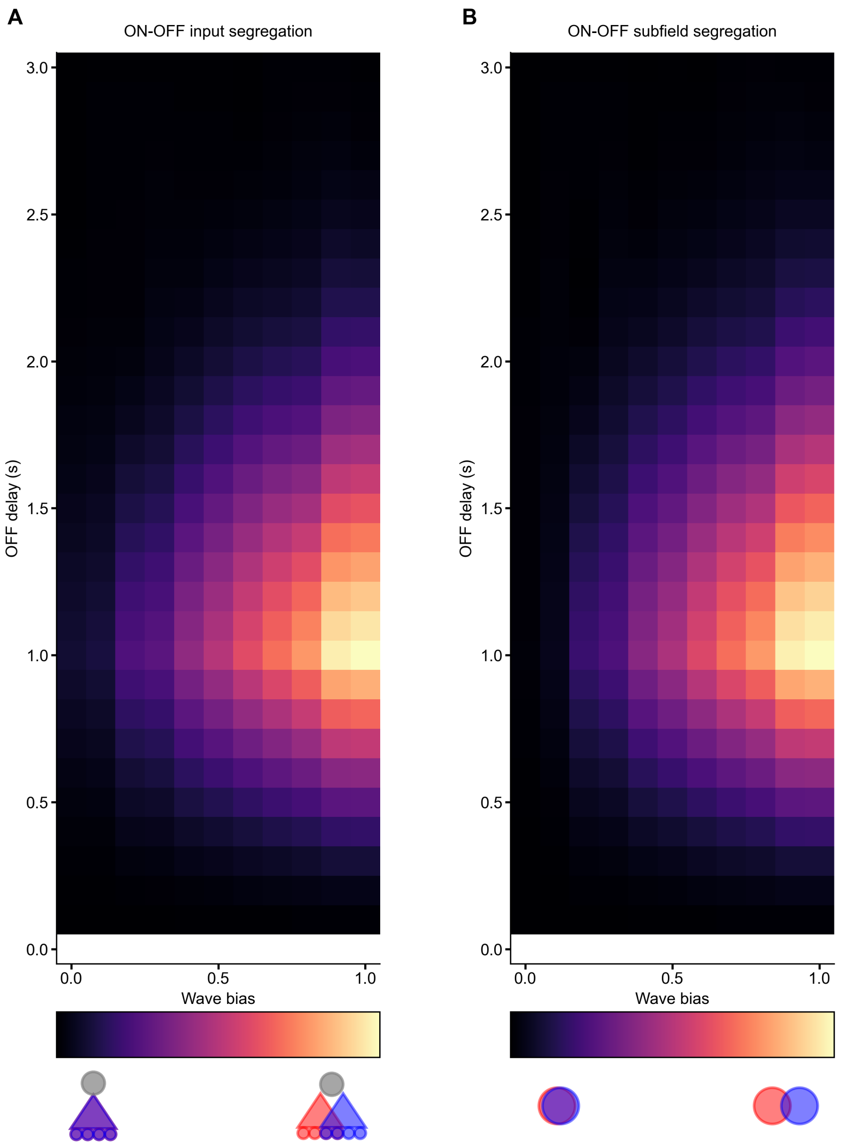

Figure S4: The ON-OFF input segregation reflects the ON-OFF subfield segregation. (A-B) The ON-OFF input segregation (A) is highly correlated to the ON-OFF subfield segregation (B). The arbor diameter used was 250 μm.

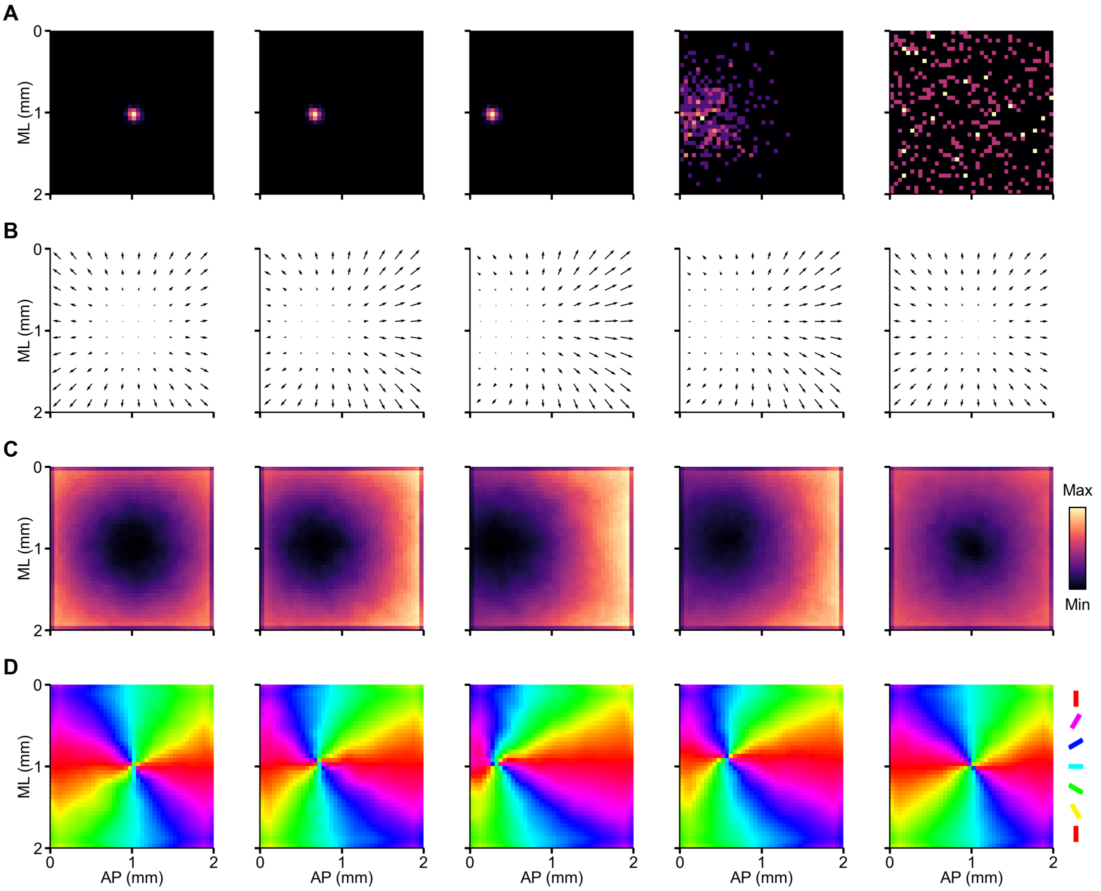

Figure S5: Effects of asymmetric inhibition center and wave initiation map on the wave flow and orientation map. (A) The asymmetric inhibition distribution with spreads σ^{AI} of $\{50, 50, 50, 350, 2000\}\mu$m and center $\begin{bmatrix}\alpha_{\mathrm{AP}} \\ \alpha_{\mathrm{ML}}\end{bmatrix}$ at $\left\{\begin{bmatrix}1000 \\ 1000\end{bmatrix}, \begin{bmatrix}650 \\ 1000\end{bmatrix}, \begin{bmatrix}250 \\ 1000\end{bmatrix}, \begin{bmatrix}250 \\ 1000\end{bmatrix}, \begin{bmatrix}250 \\ 1000\end{bmatrix}\right\}\mu$m. (B-D) The corresponding wave flows (B), wave flow strengths (C), and orientation map (D) produced with the asymmetric inhibition distribution in A. Note that the orientation map produced with $\sigma^{\mathrm{AI}} = 50\ \mu$m with center at $\begin{bmatrix}1000 \\ 1000\end{bmatrix}\mu$m (leftmost column) is highly similar to the orientation map produced with $\sigma^{\mathrm{AI}} = 2000\ \mu$m with center at $\begin{bmatrix}250 \\ 1000\end{bmatrix}\mu$m (rightmost column).

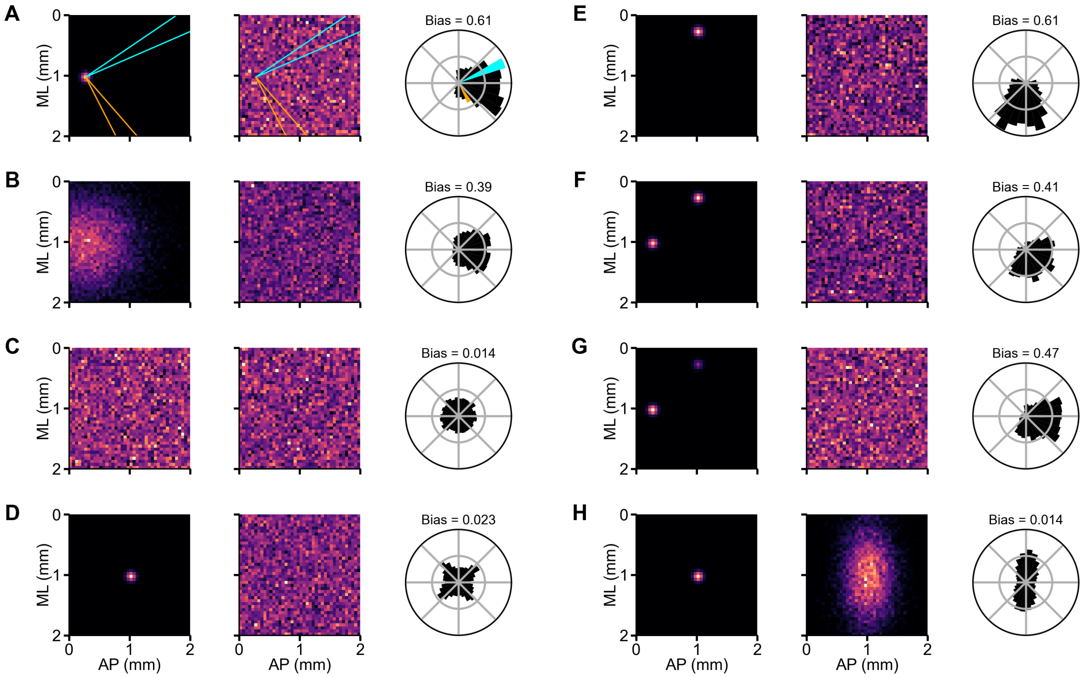

Figure S6: The wave propagation direction frequency distribution (right) can be sufficiently described by the asymmetric inhibition distribution (left) and the wave initiation map (middle). (A) Asymmetric inhibition distribution with small spread (point-like) produces a wave direction frequency distribution that corresponds to the wave initiation density covered by their respective angle intervals. This holds as long as the structural shape of the SC (not the wave initiation map, which is within the SC and can be of any shape) is convex. In the case of uniform wave initiation map, the wave direction frequency distribution approximates the shape of the wave initiation map centered at the center of asymmetric inhibition distribution. For example, the region enclosed by the cyan boundary covered more area compared to the region enclosed by the orange boundary, therefore the frequency of wave propagation directions within the cyan interval is higher than the orange interval. (B-C) As the σ^{AI} increases while keeping the center of asymmetric inhibition distribution the same as in A, the wave direction frequency distribution becomes smoother and has lower bias. This is likely due to the effect of weighted averaging the asymmetric inhibition distribution over a larger area. (D-E, H) Examples showing the asymmetric inhibition distribution centered at different locations produce wave direction frequency distribution that approximates the shape of the wave initiation map. (F-G) Examples showing the effects of weighted averaging the asymmetric distribution in A and E. Asymmetric inhibition distribution made up of two point-like centers of equal (F) and unequal (G) peaks. Their wave direction frequency distribution is the weighted average of the wave direction frequency distributions of A and E. (H) A prediction of the asymmetric inhibition distribution and wave initiation map from the wave direction frequency distribution of the FRMD7^{tm} mice (*13*).

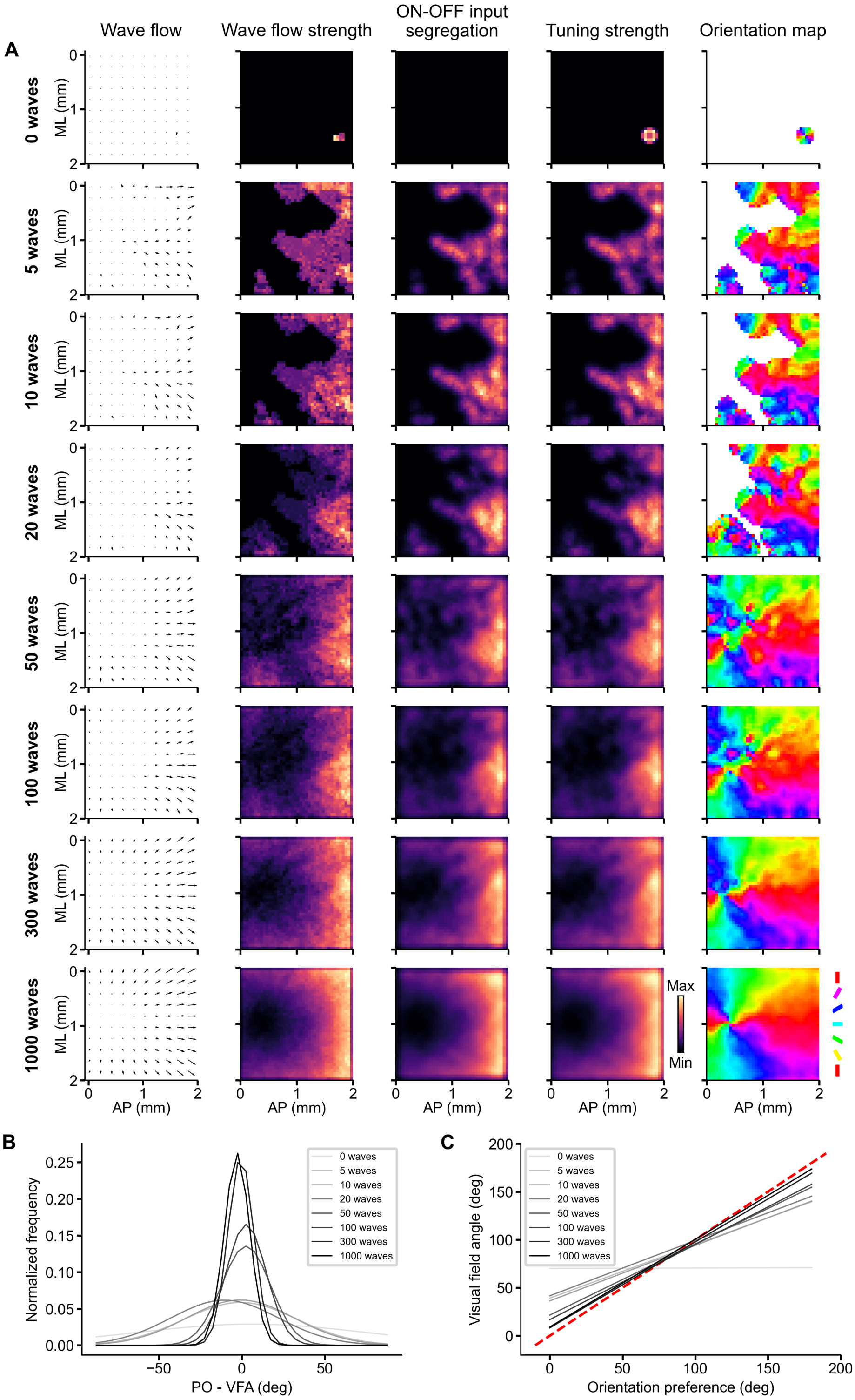

Figure S7: Development of orientation map. (A) The development of the wave flow, wave flow strengths, ON-OFF input segregation, tuning strengths of the SC RFs, and the orientation map after 0, 5, 10, 20, 50, 100, 300, and 1000 waves. (B) The distribution of the angular difference between the preferred orientation and the concentric visual field angles for different developmental stages. Over the wave iterations, the angular difference becomes smaller. (C) The fitting of concentric visual field angles against orientation preferences for different developmental stages. Over the wave iterations, the preferred orientations become more similar to the concentric visual field angles, where the red diagonal dashed line represents the perfect match between them.

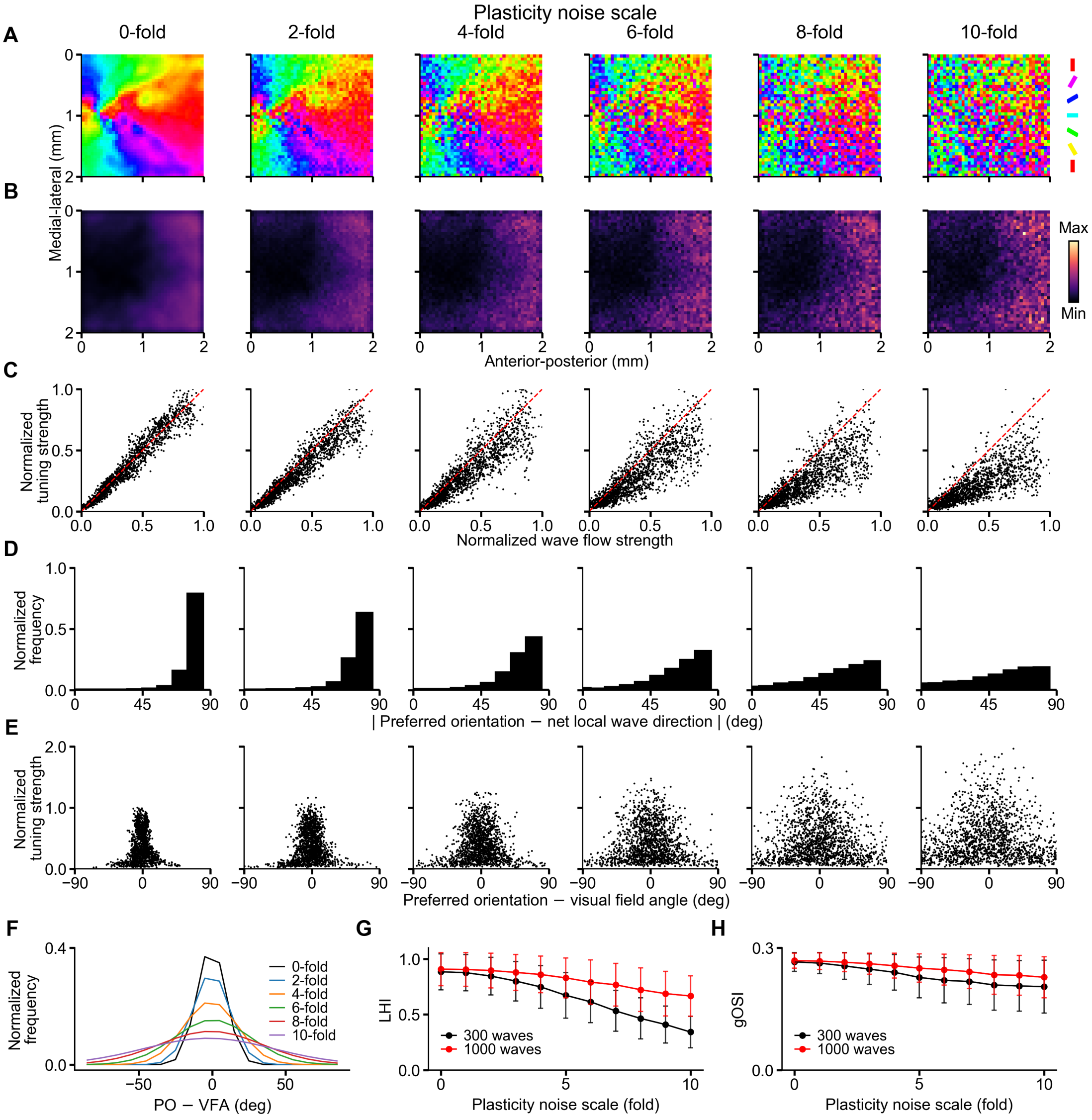

Figure S8: Robustness of the model to plasticity noise during development. (A) The orientation map developed after 300 waves with plasticity noise scales of 0-, 2-, 4-, 6-, 8-, and 10-fold. The synaptic weight update with noise at time t was computed by $\frac{d}{dt}w'_{\mathrm{P}}(\vec{x},\vec{\alpha},t) = \frac{d}{dt}w_{\mathrm{P}}(\vec{x},\vec{\alpha},t) + \gamma\xi_{\mathrm{P}}(\vec{x},\vec{\alpha},t)$, where γ is the noise scale and $\xi_{\mathrm{P}}(\vec{x},\vec{\alpha},t) \sim \mathcal{N}(0,(\frac{d}{dt}w_{\mathrm{P}}(\vec{x},\vec{\alpha},t))^2)$ is the Gaussian noise. The orientation map becomes less smooth as the plasticity noise increases. However, although the local orientation preferences become more random and exhibit a salt-and-pepper-like pattern, the global concentric pattern of the orientation map is still quite prominent up until 6-fold plasticity noise. (B) The corresponding tuning strengths of the orientation map in A. (C) The tuning strength and the wave flow strength become less and less correlated with increasing plasticity noise. (D) Increasing the plasticity noise disrupts the orthogonality between the preferred orientation and the net local wave direction. (E) The tuning strength normalized to the maximum tuning strength without plasticity noise. As the plasticity noise increases, the angular difference between the preferred orientation and the concentric visual field angle becomes larger. However, in contrast to the activity noise that decreases the tuning strength monotonically (Fig. 6C), the plasticity noise disperses the tuning strengths. (F) The distribution of the angular difference between the preferred orientation and the concentric visual field angles becomes wider as the plasticity noise increases. (G) Similar to the activity noise, the plasticity noise decreases the LHI of the orientation map. The disrupted LHI could be alleviated by increasing the number of waves, which could be explained by the law of large numbers. This could be an explanation for the increase of wave frequency from S2 to S3 (*14*). (H) Similar to the tuning strength, the gOSI is robust against the plasticity noise in general. The plasticity noise did not reduce the gOSI as much as the activity noise, but rather dispersed the gOSI. Error bars indicate the standard deviation.

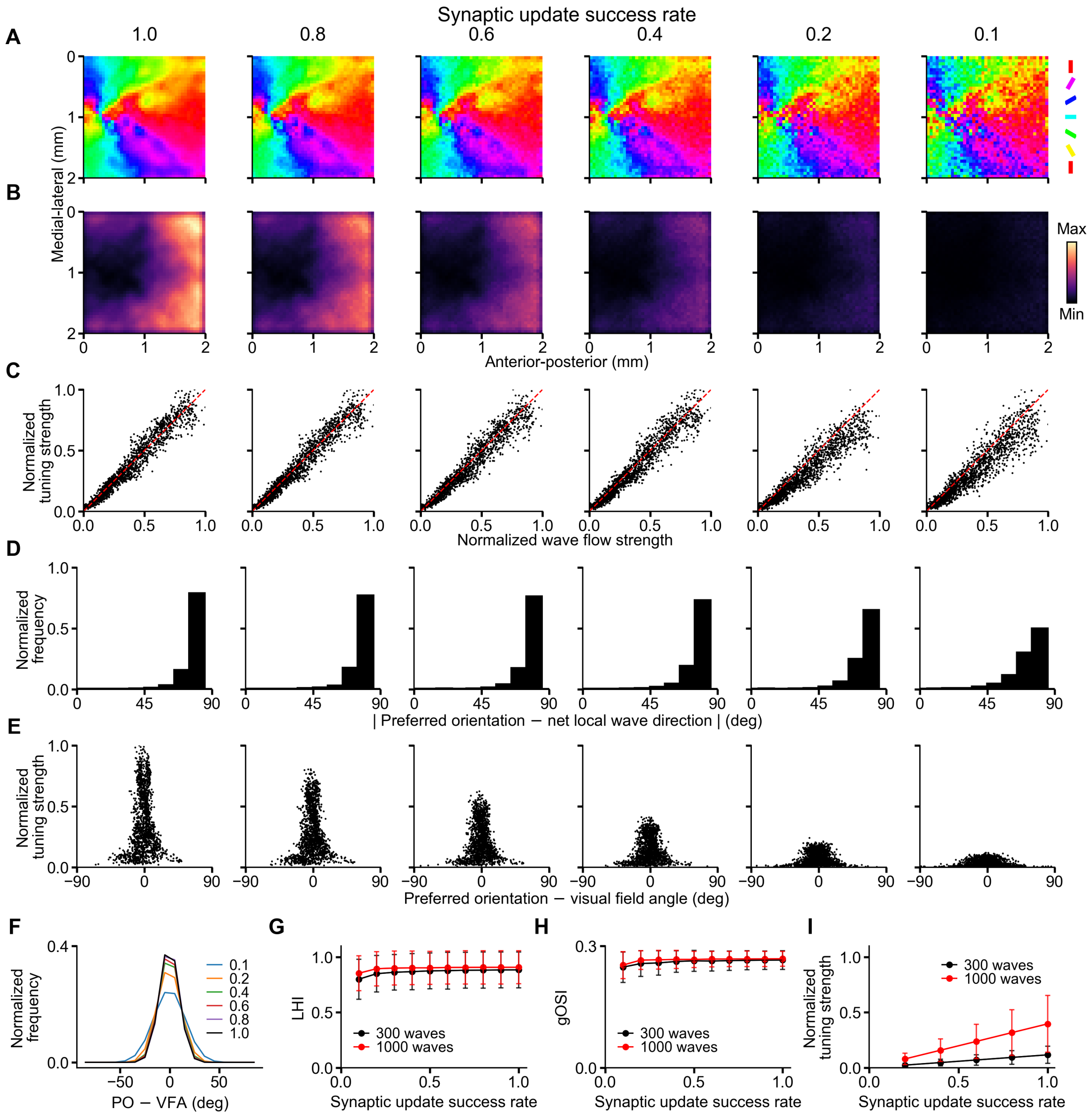

Figure S9: Robustness of the model to synaptic update failures during development. (A) The orientation map developed after 300 waves with synaptic update success rate ρ_{syn} of 1, 0.8, 0.6, 0.4, 0.2, and 0.1. For every weight update iteration, a sample for each synapse was drawn from a binomial distribution with the success rate ρ_{syn} to determine whether to update the synapse or not. The global orientation map structure hardly gets affected even if there are only 10% synapses get updated. (B) The corresponding tuning strengths of the orientation map in A. The tuning strengths were greatly reduced by the synaptic update failures. (C) The correlation between tuning strength and the wave flow strength was largely unaffected by the synaptic update failures. (D) The orthogonality between the preferred orientation and the net local wave direction is robust against the dropouts of the synaptic update. (E) The tuning strength normalized to the maximum tuning strength without synaptic update failures. As the synaptic update failure increases, the tuning strength decreases drastically, similar to the effect by the activity noise (Fig. 6C). However, in contrast to the effects by the activity noise and the plasticity noise (Fig. S8E), the angular difference between the preferred orientation and the concentric visual field angle does not get affected substantially. (F) The distribution of the angular difference between the preferred orientation and the concentric visual field angles remains largely unchanged as the synaptic update failure increases. (G) Since the overall orientation map organization is not disrupted by decreasing the synaptic update success rate, the LHIs of the map developed with 1000 waves did not improve much compared to the map developed with 300 waves. (H) Similar to the LHI, the gOSI is also robust against synaptic update failures. The synaptic update dropouts barely affected the gOSI. (I) The tuning strength normalized to the maximum tuning strength of a map developed with 1000 waves without synaptic update failures. The tuning strength increases with the synaptic update success rate as well as the number of waves. This suggests that a larger wave number can rescue the detrimental effects of synaptic update failures on the tuning strength. Error bars indicate the standard deviation.

Data S1: Data and code for plotting the *in vivo* results. The data files containing *in vivo* neural activities from visual layers of the mouse SC recorded extracellularly with high-density-electrode Neuropixels probes and the corresponding code for accessing the data and plotting the results in Fig. 1B, Fig. 2A, and Fig. S1.
